# Comparative efficacy of different repetitive transcranial magnetic stimulation protocols for lower extremity motor function in stroke patients: a network meta-analysis

**DOI:** 10.3389/fnins.2024.1352212

**Published:** 2024-02-15

**Authors:** Chengshuo Wang, Qin Zhang, Linli Zhang, Dongyan Zhao, Yanan Xu, Zejian Liu, Chunli Wu, Shengzhu Wu, Mingjin Yong, Liang Wu

**Affiliations:** ^1^Tianjin Key Laboratory of Exercise Physiology and Sports Medicine, Institute of Sport, Exercise and Health, Tianjin University of Sport, Tianjin, China; ^2^Beijing Xiaotangshan Hospital, Beijing, China; ^3^Department of Rehabilitation Medicine, Neck-Shoulder and Lumbocrural Pain Hospital of Shandong First Medical University, Jinan, China; ^4^Department of Rehabilitation, Lianyungang Hospital of Traditional Chinese Medicine, Lianyungang, China

**Keywords:** stroke, lower extremity, motor function, repetitive transcranial magnetic stimulation, network meta-analysis

## Abstract

**Background:**

Lower extremity motor dysfunction is one of the most severe consequences after stroke, restricting functional mobility and impairing daily activities. Growing evidence suggests that repetitive transcranial magnetic stimulation (rTMS) can improve stroke patients’ lower extremity motor function. However, there is still controversy about the optimal rTMS protocol. Therefore, we compared and analyzed the effects of different rTMS protocols on lower extremity motor function in stroke patients using network meta-analysis (NMA).

**Methods:**

We systematically searched CNKI, WanFang, VIP, CBM, PubMed, Embase, Web of Science, and Cochrane Library databases (from origin to 31 December 2023). Randomized controlled trials (RCTs) or crossover RCTs on rTMS improving lower extremity motor function in stroke patients were included. Two authors independently completed article screening, data extraction, and quality assessment. RevMan (version 5.4) and Stata (version 17.0) were used to analyze the data.

**Results:**

A total of 38 studies with 2,022 patients were eligible for the NMA. The interventions included HFrTMS-M1, LFrTMS-M1, iTBS-Cerebellum, iTBS-M1, dTMS-M1, and Placebo. The results of NMA showed that LFrTMS-M1 ranked first in FMA-LE and speed, and HFrTMS-M1 ranked first in BBS, TUGT, and MEP amplitude. The subgroup analysis of FMA-LE showed that HFrTMS-M1 was the best stimulation protocol for post-stroke time > 1 month, and LFrTMS-M1 was the best stimulation protocol for post-stroke time ≤ 1 month.

**Conclusion:**

Considering the impact of the stroke phase on the lower extremity motor function, the current research evidence shows that HFrTMS-M1 may be the preferred stimulation protocol to improve the lower extremity motor function of patients for post-stroke time > 1 month, and LFrTMS-M1 for post-stroke time ≤ 1 month. However, the above conclusion needs further analysis and validation by more high-quality RCTs.

**Systematic Review Registration:**www.crd.york.ac.uk/prospero/, identifier (CRD42023474215).

## 1 Introduction

Stroke is a local brain dysfunction caused by acute cerebrovascular disease (Park et al., 2011). By 2019, the proportion of stroke patients had increased to 2.58% among residents aged ≥40 years in China (Wang et al., 2022). Stroke is the leading cause of adult death and disability in China, with the five characteristics of high incidence, high disability rate, high mortality rate, high recurrence rate, and high economic burden, which has seriously endangered the health of Chinese people. With the development of medical technology, the mortality rate of stroke has decreased year by year, but 72% of the survivors still have lower extremity dysfunction (Ng and Hui-Chan, 2010), which affects the walking function of patients. Walking dysfunction is one of the most severe consequences of stroke. Nearly 30% of stroke patients are unable to walk even in the chronic recovery stage (Park et al., 2011), which significantly affects the patients’ social interactions and can lead to lifelong disability in severe cases (Park and An, 2016). Therefore, improving the motor function of the lower extremity, restoring independent walking as soon as possible, and improving activities of daily living (ADL) are the problems that many stroke patients are eager to solve urgently. However, pharmacological therapy (Mead et al., 2013) and traditional rehabilitation therapies [e.g., neurodevelopmental therapy (Langhammer and Stanghelle, 2011), proprioceptive neuromuscular facilitation (Eng and Tang, 2007), and electromyography biofeedback (Woodford and Price, 2007)] seem to have limited effects on improving motor function of the lower extremity after stroke. Therefore, a more effective treatment is needed.

Repetitive transcranial magnetic stimulation (rTMS), one of the brain stimulation techniques without any trauma, can induce neuroplastic changes and promote brain function restoration (Nathou et al., 2018). At present, the interhemispheric competition (IHC) model is the primary theoretical basis for applying rTMS in stroke rehabilitation. This model suggests that stroke destroys the balance of mutual inhibition of the bilateral cerebral hemispheres through the corpus callosum, resulting in the decreased inhibition of the ipsilateral hemisphere to the contralateral hemisphere and the increased inhibition of the contralateral hemisphere to the ipsilateral hemisphere (Di Pino et al., 2014). Therefore, in clinical practice, there are two main ways to use rTMS to promote functional recovery after stroke. One is to reduce the excitability of the contralateral hemisphere through low-frequency (≤1 Hz) rTMS to reduce the inhibitory effect of the contralateral hemisphere on the ipsilateral hemisphere. The other is to restore the balance of competitive inhibition between the bilateral cerebral hemispheres by stimulating the ipsilateral hemisphere with high-frequency (≥5 Hz) rTMS to increase its excitability (George and Aston-Jones, 2010). Both stimulation modes have been used to treat motor/non-motor dysfunction after stroke.

Theta burst stimulation (TBS), a novel mode of rTMS, saves time in the rehabilitation of motor function after stroke (Huang et al., 2005). There are two types of TBS: intermittent TBS (iTBS) and continuous TBS (cTBS), which generate excitatory and inhibitory effects, respectively (Larson et al., 1986; Huang et al., 2011). Compared with conventional rTMS protocols, TBS provides significant advantages due to its reduced stimulation time (Chung et al., 2015) and long-lasting effects with lower-intensity stimulation (Cárdenas-Morales et al., 2010). Deep transcranial magnetic stimulation (dTMS) is a new non-invasive neuromodulation technique based on rTMS technology, which uses a different coil type (Hesed coil). dTMS has the advantages of deeper and wider stimulation, more precise localization, and less damage to the superficial cortex than conventional TMS (Roth et al., 2014). The primary motor cortex (M1) is typically the target of rTMS. However, some studies have shown that the cerebellum is one of the alternative targets of M1, and rTMS targeting the cerebellum can also improve motor function in stroke patients (Wessel and Hummel, 2018). In conclusion, rTMS can regulate the asymmetry of excitability between the bilateral cerebral hemispheres by changing the stimulation mode, stimulation target, stimulation frequency, and coil type to promote the recovery of lower extremity motor dysfunction after stroke (Kesikburun, 2022).

The effect of rTMS on lower extremity motor function in stroke patients has been demonstrated in previous meta-analysis (Li et al., 2018; Tung et al., 2019). However, an important drawback is that conventional meta-analysis can only compare two interventions simultaneously. At the same time, in these studies, the intervention protocols of the experimental group were roughly classified, and the effects of different stimulation frequencies, stimulation targets, stimulation modes, and post-stroke times on treatment effects were not comprehensively considered. Although Fan et al. (2021) conducted a detailed systematic review of rTMS to improve lower extremity motor function in stroke patients, this study did not consider the new stimulation mode-iTBS, the new stimulation target-cerebellum, and the effect of stroke phase on the efficacy of rTMS.

Network meta-analysis (NMA) is developed from conventional meta-analysis, and its primary function is to comprehensively evaluate and rank multiple interventions simultaneously (Rouse et al., 2017). Therefore, we took the stimulation mode, stimulation frequency, and stimulation target of rTMS into account and summarized the following five different rTMS protocols: high-frequency rTMS-M1 (HFrTMS-M1), low-frequency rTMS-M1 (LFrTMS-M1), iTBS-Cerebellum, iTBS-M1, and dTMS-M1. Then, we compared and analyzed the effects of different protocols on lower extremity motor dysfunction in stroke patients by NMA. In addition, considering the effects of the stroke phase on the efficacy of rTMS at different protocols, we also carried out a subgroup analysis for FMA-LE according to the phase of the stroke to provide sufficient evidence for future clinical practice.

## 2 Materials and methods

### 2.1 Study enrollment and reporting

Our NMA was conducted using the Cochrane Handbook for Systematic Reviews of Interventions, and the findings were reported according to the Preferred Reporting Items for Systematic Reviews and Meta-Analyzes (PRISMA) statement (Page et al., 2021). This NMA was prospectively registered in PROSPERO (registration ID: CRD42023474215).

### 2.2 Search strategy

Two authors separately searched for randomized controlled trials (RCTs) and crossover RCTs about rTMS improving lower extremity motor function in stroke patients from China National Knowledge Infrastructure (CNKI), Wanfang database, VIP database, China Biomedical Literature Database (CBM), PubMed, Embase, Web of Science, and Cochrane Library. The search time limit was from the establishment of the database to 31 December 2023. By combining medical subject headings (MeSH) with free words using Boolean logic operators, we integrated the following terms for a comprehensive search: “Stroke,” “cerebrovascular accident,” “CVA,” “Brain Vascular Accident,” “hemiplegia,” “apoplexy,” “hemiparesis,” “repetitive transcranial magnetic stimulation,” “Transcranial Magnetic Stimulation,” “TMS,” “rTMS,” “Theta burst stimulation,” “θ-brust stimulation,” “random,” “randomized controlled trial,” “RCT.” In addition, we also reviewed meta-analysis, reviews, and references of the included studies to supplement the search. PubMed was used as an example, and the specific search strategy was provided in Supplementary Table 1.

### 2.3 Eligibility criteria

Eligibility criteria were defined in accordance with the PICOS framework (Hutton et al., 2015).

#### 2.3.1 Inclusion criteria

1. Populations: Stroke patients with lower extremity dysfunction who were diagnosed according to the stroke diagnostic criteria formulated by The Fourth National Cerebrovascular Disease Conference in 1995.
2. Interventions: HFrTMS-M1, LFrTMS-M1, iTBS-Cerebellum, iTBS-M1, and dTMS-M1.
3. Comparators: The placebo included conventional rehabilitation and sham rTMS (or conventional rehabilitation alone). Sham rTMS refers to the analog sound without any effective magnetic stimulation. Conventional rehabilitation, such as physiotherapy, occupational therapy, physical therapy, treadmill training, motor imagery practice, task-oriented training, and mirror therapy, was acceptable as cointervention.
4. Outcomes: The primary outcome indicator was the Fugl-Meyer Assessment of Lower Extremity (FMA-LE). The secondary outcome indicators included the Berg Balance Scale (BBS), Timed Up and Go Test (TUGT), Motor Evoked Potential amplitude (MEP amplitude), and speed.
5. Study designs: RCTs or crossover RCTs.

#### 2.3.2 Exclusion criteria

1. Patients with lower extremity motor dysfunction were not caused by stroke but by traumatic brain injury, cerebral palsy, Parkinson’s disease, and other diseases.
2. Conference abstracts, researcher protocols, reviews, meta-analysis, dissertations, and non-RCTs (e.g., case reports, observational studies, cross-sectional studies, and studies without a control group).
3. Lack of outcome indicators related to the lower extremity motor function.
4. Studies with more patients withdrawing midway.
5. Studies that could not be downloaded.
6. Studies with incomplete outcome data and contacting the authors three times without response.
7. Repeatedly published studies.

### 2.4 Study selection

First, two authors (CSW and QZ) used EndnoteX9 software to eliminate duplicate articles. Then, they screened out articles that did not meet the criteria by reading their titles and abstracts. Finally, they browsed the full text to select articles that met the criteria. In case of any disagreement during the review process, the decision was made by consultation between the two authors or by joint decision with the third author (LLZ).

### 2.5 Data extraction

Two authors (DYZ and YNX) independently reviewed all articles and extracted data. The extracted data included basic published information: first author’s name, year of publication, country of origin, participant characteristics (age and sample size), intervention characteristics (intervention protocol, coil type, rTMS target, rTMS frequency, rTMS intensity, No. of pulses, and duration of intervention), and outcome indicators (FMA-LE, BBS, TUGT, MEP amplitude, and speed) at baseline and at last observation to obtain their change scores. The collected data were put into an Excel spreadsheet and cross-checked by two authors (DYZ and YNX). In case of disagreement during data extraction, the third author (ZJL) participated in discussion and decision-making.

### 2.6 Quality assessment

Two authors (CLW and SZW) independently assessed the risk of bias for the included articles through the Cochrane Risk of Bias Tool (Savović et al., 2014), which mainly included seven indicators: (I) Random sequence generation; (II) Allocation concealment; (III) Blinding of participants and personnel; (IV) Blinding of outcome assessment; (V) Incomplete outcome data; (VI) Selective reporting; (VII) Other bias. Assessment indicators were rated “low risk,” “unclear,” or “high risk” based on the available information. If there was any dispute during the evaluation, the third author (ZJL) would participate in the discussion and make decisions together.

### 2.7 Statistical analysis

Odds ratio (OR) for binary variables and mean difference (MD) for continuous variables were used as the effect indicators, and the 95% confidence interval (CI) was provided for each effect size. If a particular study used different methods or scales to measure the same outcome, standardized MD (SMD) was calculated instead of MD. We calculated the difference before and after treatment for continuous variable indicators and the standard deviation (SD) according to the method provided in 16.1.3.2 of Cochrane Handbook 5.0.2 and then performed the statistical analysis. We used RevMan (version 5.4) for pairwise meta-analysis. The *p*-value of the chi-square test and the I^2^ index from the heterogeneity test were used to express the level of statistical heterogeneity. Different effect models were selected according to the test data’s heterogeneity level. When the level of heterogeneity was low (*p* ≥ 0.1, I^2^ ≤ 50%), we selected the fixed effect model for analysis. Otherwise, a random effect model (*p* < 0.1, I^2^ > 50%) was used (Higgins et al., 2003; Tufanaru et al., 2015).

We used Stata (version 17.0) to perform the NMA and produce various charts, such as network meta-analysis diagrams of eligible comparisons, surface under the cumulative ranking area (SUCRA), funnel plot of publication bias, etc (Shim et al., 2017). When there were closed loops between interventions, we first needed to assess global inconsistency. If *p* > 0.05, the inconsistent model was not significant, and the consistent model was selected (White et al., 2012). We used a node-splitting approach to assess local inconsistency (Dias et al., 2010). At the same time, it was also necessary to evaluate the loop inconsistency and calculate the inconsistency factors (IF) and 95% CI for each closed loop. If the lower limit of 95% CI included or was close to 0, the consistency between the direct and indirect comparison results was good; otherwise, the closed loop was considered to have apparent inconsistency. If no closed loops existed between interventions, the consistency model was used directly for analysis. We used the SUCRA to rank interventions. The closer SUCRA was to 100%, the better the effect of the intervention. Finally, the publication bias of the included articles was evaluated using the funnel plot of publication bias and Egger’s test. Asymmetry in the funnel plot of publication bias and *p* < 0.05 in Egger’s test indicated publication bias in the included articles (Fleiss, 1993).

## 3 Results

### 3.1 Search results

**Figure 1:**
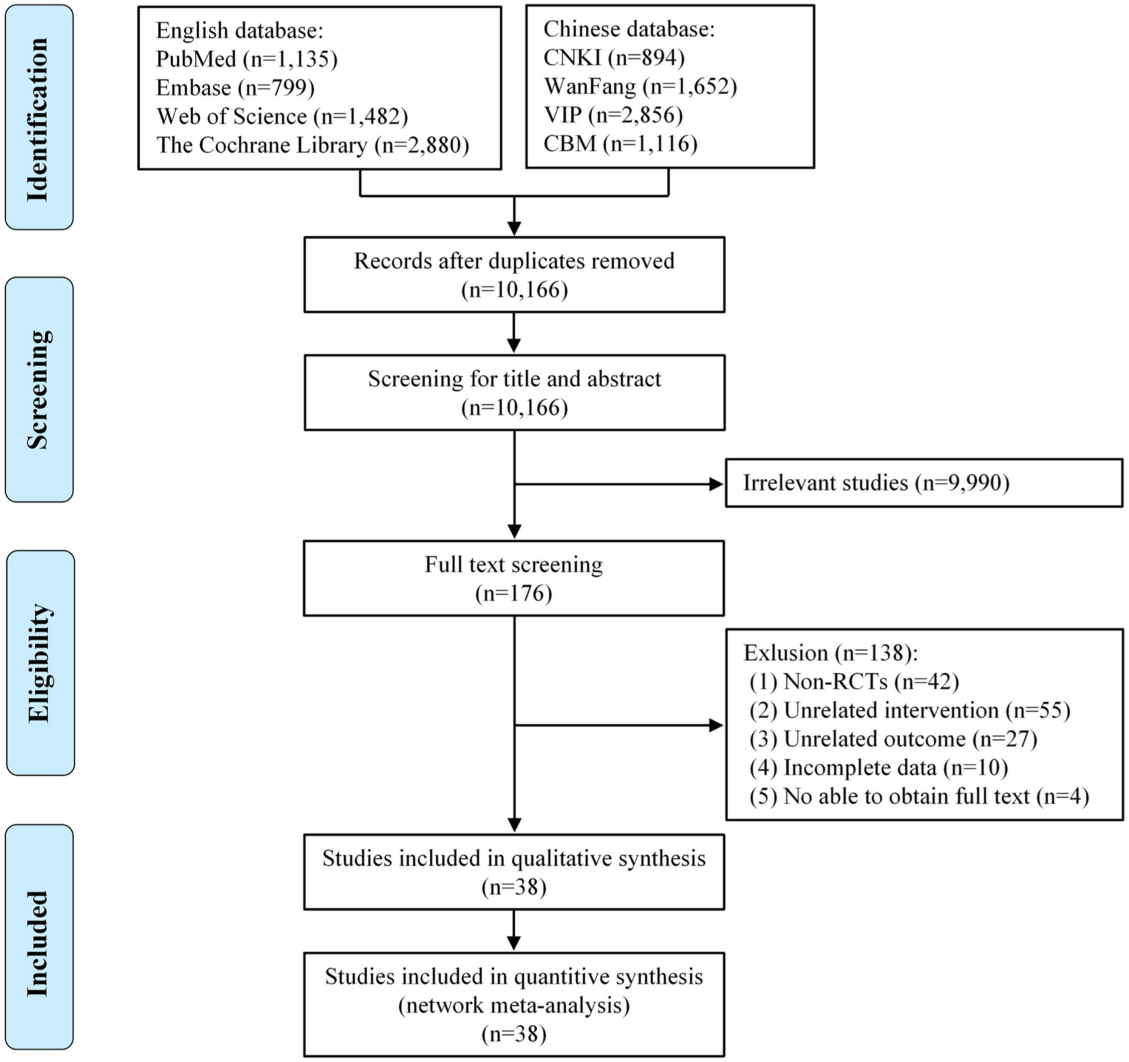
Flow diagram of the eligible studies selection process. CNKI, China National Knowledge Infrastructure; WanFang, WanFang Knowledge Service Platform; VIP, Chinese Scientific Journals Database; CBM, Chinese Biomedical Literature Service System; n, number of publications.

We strictly searched the above 8 databases according to the inclusion and exclusion criteria and preliminarily obtained 12,814 articles. After the duplicate articles were removed, 10,166 articles remained in the database. By reading the titles and abstracts of the articles, we excluded articles that did not meet the inclusion criteria, leaving 176 remaining in the database. By reading the full text, we again excluded 138 articles. Ultimately, 38 articles met our study requirements. Figure 1 shows the article search process and results.

### 3.2 Characteristics of the included studies

Characteristics of studies adopted are shown in Table 1, published between 2012 and 2023. We finally included 38 studies with a total of 2,022 patients. 4 studies were crossover RCTs, and 34 studies were RCTs. Among the included studies, most of them were carried out in China (28/38), and the others were conducted in Korea (5/38), Italy (2/38), Egypt (1/38), India (1/38), and Japan (1/38). Outcome indicators included FMA-LE (29 studies), BBS (11 studies), TUGT (8 studies), MEP amplitude (8 studies), and speed (13 studies). Interventions included HFrTMS-M1 (16 studies), LFrTMS-M1 (18 studies), iTBS-Cerebellum (5 studies), iTBS-M1 (1 study), and dTMS-M1 (2 studies).

**Table 1:** Characteristics of included studies.

Study	Country	Age (E/C, year)	Sample size (E/C)	Intervention							Outcomes
				Intervention protocol (E/C)	Coil type	rTMS target	rTMS frequency (Hz)	rTMS intensity (%)	No. of pulses	Duration of intervention	
Yang et al. (2016)	China	56.4 ± 7.8/57.5 ± 9.2	14/14	HFrTMS-M1/Placebo	75-mm figure-of-8 coil	Ipsi-M1	10 Hz	90% RMT	2,000	6 times per week for 4 weeks	①⑤
Ma et al. (2023)	China	56.75 ± 7.07/54.85 ± 5.65	20/20	HFrTMS-M1/Placebo	NR	Ipsi-M1	10 Hz	80% RMT	1,200	5 times per week for 4 weeks	①③⑤
Mo and Liu (2020)	China	56.68 ± 3.12/57.75 ± 2.86	53/52	HFrTMS-M1/Placebo	Figure-of-8 coil	Ipsi-M1	10 Hz	90% RMT	2,000	7 times per week for 4 weeks	①②⑤
Cha et al. (2014)	Korea	54.83 ± 6.32/51.33 ± 8.71	12/12	HFrTMS-M1/LF-ZrTMS	70-mm figure-of-8 coil	Ipsi-M1/Contra-M1	10 Hz/1 Hz	90% RMT	2,000/1,200	5 times per week for 4 weeks	②④
Cha and Kim (2017)	Korea	53.80 ± 13.28/55.80 ± 16.40	10/10	HFrTMS-M1/Placebo	70-mm figure-of-8 coil	Ipsi-M1	10 Hz	90% RMT	1,000	5 times per week for 8 weeks	④⑤
Wang et al. (2019)	China	53.5 ± 13.7/54.7 ± 12.2	8/6	HFrTMS-M1/Placebo	Figure-of-8 coil	Ipsi-M1	5 Hz	90% RMT	900	3 times per week for 3 weeks	①④⑤
Lee and Cha (2020)	Korea	66.85 ± 4.05/64.00 ± 3.57	7/6	HFrTMS-M1/Placebo	70-mm figure-of-8 coil	Ipsi-M1	5 Hz	90% RMT	900	5 times per week for 3 weeks	③
Ji et al. (2014)	Korea	49.00 ± 11.01/44.28 ± 8.52	15/15	HFrTMS-M1/Placebo	70-mm figure-of-8 coil	Ipsi-M1	10 Hz	NR	NR	3 times per week for 6 weeks	⑤
Ji and Kim (2015)	Korea	55.65 ± 8.95/56.36 ± 10.44	20/19	HFrTMS-M1/Placebo	70-mm figure-of-8 coil	Ipsi-M1	10 Hz	NR	2,000	5 times per week for 4 weeks	⑤
Ceng et al. (2022)	China	60.22 ± 2.73/61.41 ± 2.24	40/40	HFrTMS-M1/Placebo	NR	Ipsi-M1	10 Hz	80% RMT	NR	5 times per week for 8 weeks	①
Kakuda et al. (2013)	Japan	52.1 ± 11.9	9/9	HFrTMS-M1/Placebo	80-mm double-cone coil	Bi-M1	10 Hz	90% RMT	2,000	1 time	⑤
Guan et al. (2017)	China	59.7 ± 6.8/57.4 ± 14.0	21/21	HFrTMS-M1/Placebo	Figure-of-8 coil	Ipsi-M1	5 Hz	120% RMT	1,000	10 consecutive weekdays	①
Wang et al. (2023)	China	63.85 ± 9.54/63.92 ± 10.28/ 64.10 ± 9.96	80/80/80	HFrTMS-M1/LFrTMS-M1/Placebo	NR	Ipsi-M1/Contra-M1	10 Hz/0.5 Hz	NR	NR	6 times per week for 3 weeks	①
Hua et al. (2019)	China	63.2 + 9.5/63.4 + 10.7/65.4 + 10.8	15/15/15	HFrTMS-M1/LFrTMS-M1/Placebo	Figure-of-8 coil	Ipsi-M1/Contra-M1	10 Hz/0.5 Hz	80% RMT	1,290/1,090	6 times per week for 3 weeks	①②
Liu et al. (2019)	China	58.78 ± 6.97/60.78 ± 6.73/58.44 ± 5.94	18/18/18	HFrTMS-M1/LFrTMS-M1/Placebo	NR	Ipsi-M1/Contra-M1	10 Hz/0.5 Hz	90% MT	NR	5 times per week for 3 weeks	①
Du et al. (2019)	China	54 ± 12/56 ± 9/56 ± 11	20/20/20	HFrTMS-M1/LFrTMS-M1/Placebo	90-mm figure-of-8 coil	Ipsi-M1/Contra-M1	10 Hz/1 Hz	100% RMT	1,200/1,200	5 consecutive weekdays	④
Sharma et al. (2020)	India	54.85 ± 13.39/ 52.89 ± 14.95	47/49	LFrTMS-M1/Placebo	70-mm figure-of-8 coil	Contra-M1	1 Hz	110% RMT	750	5 times per week for 2 weeks	①
Zhao et al. (2018)	China	56.2 ± 12.7/54.0 ± 11.4	36/39	LFrTMS-M1/Placebo	Circular coil	Contra-M1	1 Hz	80–120% RMT	1,000	20 consecutive weekdays	①②
Zhou et al. (2020)	China	58.80 ± 7.58/58.32 ± 7.61	50/50	LFrTMS-M1/Placebo	70-mm double-cone coil	Contra-M1	1 Hz	70% RMT	1,200	5 times per week for 3 weeks	①
Li et al. (2016)	China	56.7 ± 6.0/58.0 ± 6.5	30/30	LFrTMS-M1/Placebo	70-mm figure-of-8 coil	Contra-M1	1 Hz	90% RMT	NR	5 times per week for 4 weeks	①⑤
Yan et al. (2023)	China	67.82 ± 9.97/69.11 ± 10.03	88/88	LFrTMS-M1/Placebo	Circular coil	Contra-M1	1 Hz	70% RMT	NR	5 times per week for 6 weeks	①②⑤
Mou et al. (2021)	China	52.10 ± 14.96/48.40 ± 15.58	20/20	LFrTMS-M1/Placebo	Circular coil	Contra-M1	1 Hz	90% RMT	1,000	6 times per week for 6 weeks	①
Huang et al. (2018)	China	62.2 ± 10.4/61.2 ± 9.4	18/20	LFrTMS-M1/Placebo	110-mm double-cone coil	Contra-M1	1 Hz	120% RMT	900	15 consecutive weekdays	①③④
Lin et al. (2015)	China	58.3 ± 10.8/62.3 ± 11.7	16/16	LFrTMS-M1/Placebo	70-mm figure-of-8 coil	Contra-M1	1 Hz	130% MT	900	15 consecutive weekdays	①
Chen (2018)	China	55.2 ± 11.5/51.3 ± 12.1	70/70	LFrTMS-M1/Placebo	90-mm figure-of-8 coil	Contra-M1	1 Hz	90% RMT	1,000	5 times per week for 1 week	①②③
Wang et al. (2012)	China	64.90 ± 12.37/62.98 ± 10.88	14/14	LFrTMS-M1/Placebo	Figure-of-8 coil	Contra-M1	1 Hz	90% RMT	600	5 times per week for 2 weeks	①④⑤
Elkholy et al. (2014)	Egypt	44.06 ± 3.71/45.66 ± 4.27	30/15	LFrTMS-M1/Placebo	NR	Contra-M1	1 Hz	2 G	NR	3 times per week for 6 weeks	③⑤
Zhu et al. (2022)	China	59.48 ± 7.04/58.36 ± 5.38	25/25	LFrTMS-M1/Placebo	NR	Contra-M1	1 Hz	90% RMT	NR	5 times per week for 4 weeks	①③⑤
Tao et al. (2022)	China	57.5 ± 6.4/58.2 ± 4.8	20/20	LFrTMS-M1/Placebo	Figure-of-8 coil	Contra-M1	1 Hz	80% RMT	600	5 times per week for 4 weeks	①②
Gong et al. (2021)	China	63.40 ± 10.37/59.66 ± 14.31	16/16	LFrTMS-M1/Placebo	NR	Contra-M1	1 Hz	NR	NR	5 times per week for 4 weeks	①④
Koch et al. (2019)	China	63 ± 11/65 ± 12	18/18	iTBS-Cerebellum/Placebo	70-mm figure-of-8 coil	Contra-cerebellar	iTBS	80% RMT	1,200	3 weeks	①②
Chen et al. (2023)	China	58.88 ± 15.79/62.38 ± 12.66	16/16	iTBS-Cerebellum/Placebo	Figure-of-8 coil	Contra-cerebellar	iTBS	80% RMT	600	6 times per week for 3 weeks	①
Wang and Li (2022)	China	52.62 ± 8.61/54.62 ± 7.85	21/21	iTBS-Cerebellum/Placebo	70-mm figure-of-8 coil	Contra-cerebellar	iTBS	80% RMT	600	5 times per week for 4 weeks	①②
Liao et al. (2021)	China	51.53 ± 9.22/55.40 ± 8.10	15/15	iTBS-Cerebellum/Placebo	70-mm figure-of-8 coil	Contra-cerebellar	iTBS	90% AMT	600	5 times per week for 2 weeks	②④
Xie et al. (2021b)	China	52.35 ± 8.62/54.41 ± 7.01	18/18	iTBS-Cerebellum/Placebo	70-mm figure-of-8 coil	Contra-cerebellar	iTBS	90% AMT	600	10 consecutive weekdays	①③
Lin et al. (2019)	China	60.8 ± 8.1/61.1 ± 9.7	10/10	iTBS-M1/Placebo	70-mm figure-of-8 coil	Bi-M1	iTBS	100% RMT	1,200	2 times per week for 5 weeks	①②③
Chieffo et al. (2014)	Italy	62.20 ± 10.23	5/5	dTMS-M1/Placebo	H-coil	Bi-M1	20 Hz	90% RMT	1,500	11 times for 3 weeks	①
Chieffo et al. (2021)	Italy	58.67 ± 10.33/61.17 ± 8.70	6/6	dTMS-M1/Placebo	H-coil	Bi-M1	20 Hz	80–90% RMT	1,600	11 times for 3 weeks	①

E, experimental group; C, control group; HFrTMS, high-frequency repetitive transcranial magnetic stimulation; LFrTMS, low-frequency repetitive transcranial magnetic stimulation; iTBS, intermittent theta-burst stimulation; dTMS, deep transcranial magnetic stimulation; M1, primary motor cortex; Ipsi, ipsilateral; Contra, contralateral; Bi, bilateral; NR, not reported; MT, motor threshold; RMT, resting motor threshold; AMT, active motor threshold. ①: FMA-LE; ②: BBS; ③: TUGT; ④: MEP amplitude; ⑤: Speed.

### 3.3 Quality evaluation

**Figure 2:**
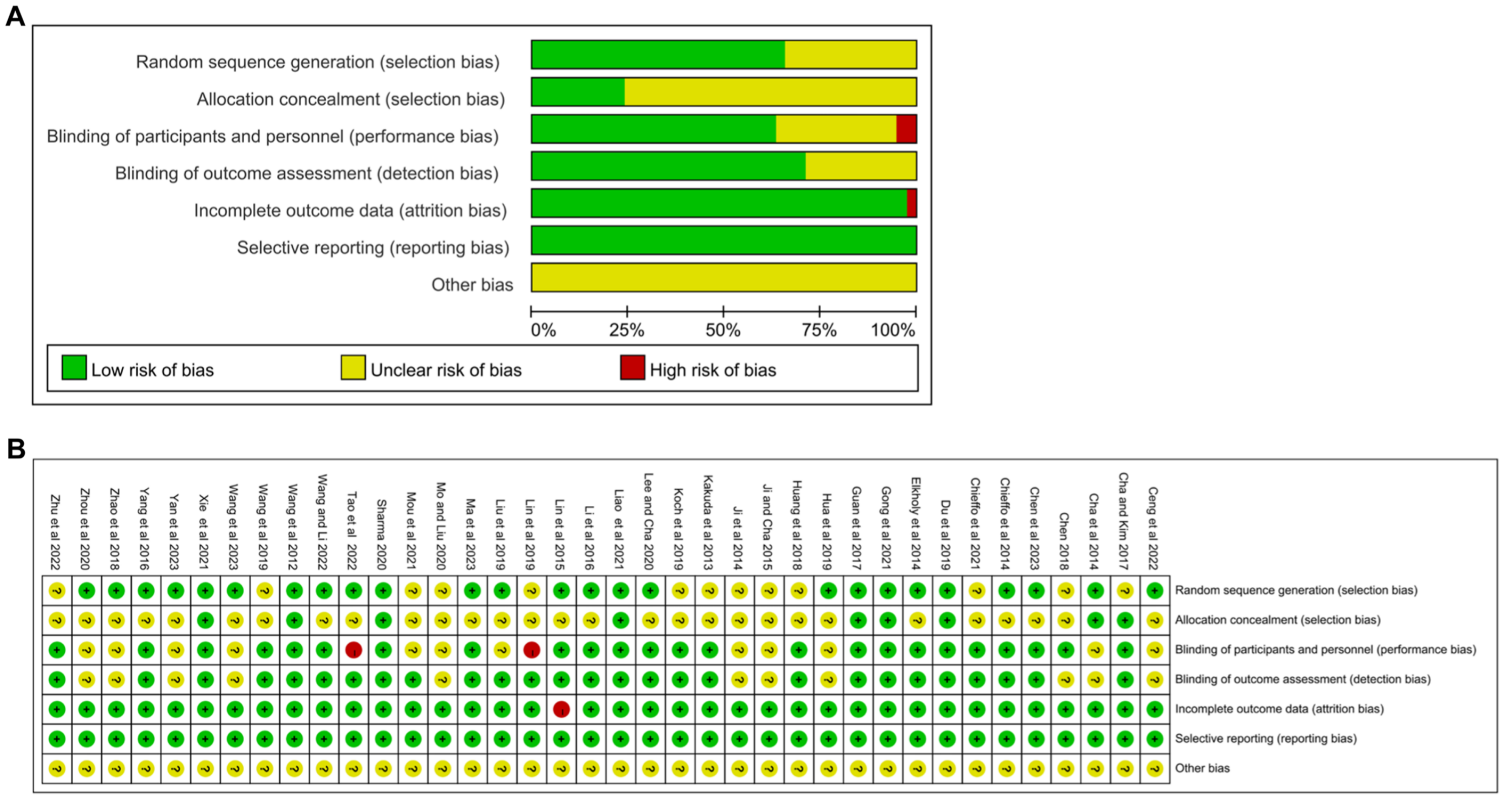
Quality assessment of selected studies by the Cochrane Risk Of Bias Tool. **(A)** Risk of bias graph: review authors’ judgments about each risk of bias item presents as percentages across all included studies. **(B)** Risk of bias summary: review authors’ judgments about each risk of bias item for each included study.

25 studies (65.8%) had a low risk of bias concerning random sequence generation. 9 studies (23.7%) had a low risk of bias concerning allocation concealment. 24 studies (63.2%) had a low risk of bias concerning blinding of participants and personnel. 27 studies (71.1%) had a low risk of bias concerning blinding of outcome assessment. 37 studies (97.4%) had a low risk of bias concerning incomplete outcome data. 38 studies (100.0%) had a low risk of bias concerning selective reporting. Other biases were not known. Details of the evaluation of bias results for the included articles are shown in Figure 2.

### 3.4 Pairwise meta-analysis

A pairwise meta-analysis was used to compare the two interventions comprehensively. We carried out 6 pairwise meta-analysis to compare FMA-LE, 6 to compare FMA-LE (post-stroke time > 1 month), 3 to compare FMA-LE (post-stroke time ≤ 1 month), 5 to compare BBS, 4 to compare TUGT, 4 to compare MEP amplitude, and 2 to compare speed, respectively, which can be summarily seen in Table 2. The detailed results of pairwise meta-analysis are shown in Supplementary Figures 1–7.

**Table 2:** Pairwise meta-analysis.

Comparison	Number of studies	MD/SMD (95% CI)	Heterogeneity test	
			I^2^ (%)	*p*-value
**FMA-LE**				
HFrTMS-M1/Placebo	9	**3.36 (2.01, 4.72)**	81	<0.00001
LFrTMS-M1/Placebo	16	**2.62 (1.71, 3.54)**	81	<0.00001
iTBS-Cerebellum/Placebo	4	0.41 (−0.74, 1.56)	0	0.88
iTBS-M1/Placebo	1	0.10 (−1.29, 1.49)	NR	NR
dTMS-M1/Placebo	2	**1.60 (0.59, 2.61)**	0	1
LFrTMS-M1/HFrTMS-M1	3	**2.37 (1.35, 3.38)**	12	32
**FMA-LE (post-stroke time > 1 month)**				
HFrTMS-M1/Placebo	6	**4.52 (2.85, 6.19)**	76	0.001
LFrTMS-M1/Placebo	9	**2.84 (1.56, 4.12)**	88	<0.00001
iTBS-Cerebellum/Placebo	4	0.41 (−0.74, 1.56)	0	0.88
iTBS-M1/Placebo	1	0.10 (−1.29, 1.49)	NR	NR
dTMS-M1/Placebo	2	**1.60 (0.59, 2.61)**	0	1
LFrTMS-M1/HFrTMS-M1	1	0.89 (−3.16, 4.94)	NR	NR
**FMA-LE (post-stroke time ≤ 1 month)**				
HFrTMS-M1/Placebo	3	**1.25 (0.44, 2.06)**	0	0.82
LFrTMS-M1/Placebo	7	**2.39 (1.16, 3.63)**	52	0.05
LFrTMS-M1/HFrTMS-M1	2	**2.47 (1.41, 3.52)**	42	0.19
**BBS**				
HFrTMS-M1/Placebo	2	**6.64 (4.37, 8.91)**	75	0.05
LFrTMS-M1/Placebo	4	**4.49 (1.75, 7.24)**	90	<0.00001
iTBS-Cerebellum/Placebo	4	**3.23 (0.99, 5.47)**	57	0.07
iTBS-M1/Placebo	1	0.60 (−1.68, 2.88)	NR	NR
HFrTMS-M1/LFrTMS-M1	2	4.34 (−5.73, 14.41)	84	0.01
**TUGT**				
HFrTMS-M1/Placebo	2	**−3.25 (−5.19, −1.30)**	37	0.21
LFrTMS-M1/Placebo	4	**−2.72 (−3.95, −1.49)**	0	0.94
iTBS-Cerebellum/Placebo	1	−0.38 (−12.70, 11.94)	NR	NR
iTBS-M1/Placebo	1	−0.70 (−4.63, 3.23)	NR	NR
**MEP amplitude**				
HFrTMS-M1/Placebo	3	**0.92 (0.08, 1.77)**	61	0.08
LFrTMS-M1/Placebo	3	0.36 (−0.48, 1.21)	78	0.01
iTBS-Cerebellum/Placebo	1	−0.10 (−0.81, 0.62)	NR	NR
HFrTMS-M1/LFrTMS-M1	2	0.71 (−0.99, 2.41)	89	0.003
**Speed**				
HFrTMS-M1/Placebo	8	**0.91 (0.68, 1.13)**	26	0.21
LFrTMS-M1/Placebo	5	**1.04 (0.81, 1.26)**	46	0.12

Red and bold numbers are statistically significant. HFrTMS, high-frequency repetitive transcranial magnetic stimulation; LFrTMS, low-frequency repetitive transcranial magnetic stimulation; iTBS, intermittent theta-burst stimulation; dTMS, deep transcranial magnetic stimulation; M1, primary motor cortex; NR, not reported.

### 3.5 Network of evidence

**Figure 3:**
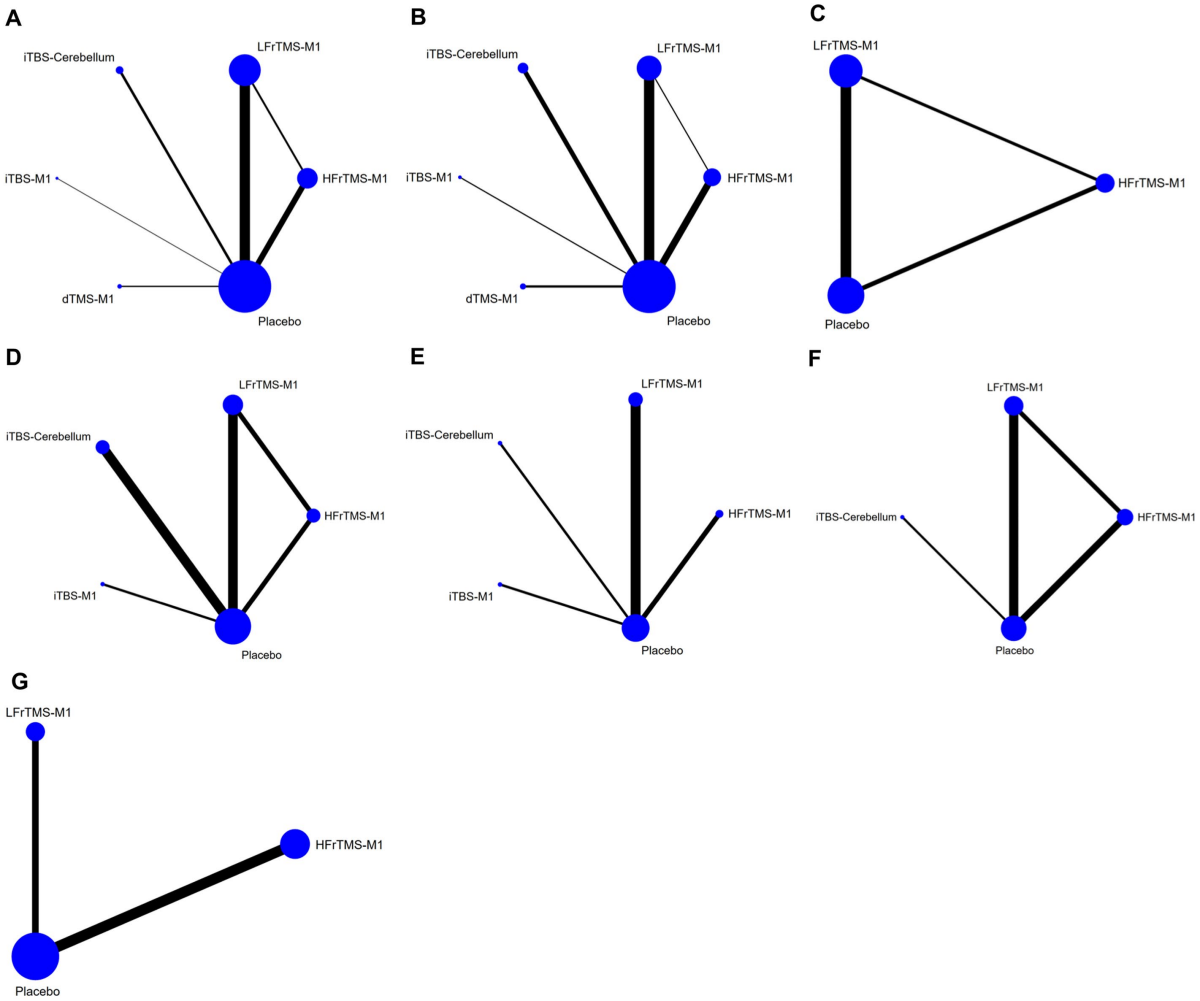
Network meta-analysis diagrams of eligible comparisons. The width of the lines is proportional to the number of trials. The size of every circle is proportional to the number of randomly assigned participants (sample size). **(A)** FMA-LE; **(B)** FMA-LE (post-stroke time > 1 month); **(C)** FMA-LE (post-stroke time ≤ 1 month); **(D)** BBS; **(E)** TUGT; **(F)** MEP amplitude; **(G)** Speed. HFrTMS, high-frequency repetitive transcranial magnetic stimulation; LFrTMS, low-frequency repetitive transcranial magnetic stimulation; iTBS, intermittent theta-burst stimulation; dTMS, deep transcranial magnetic stimulation; M1, primary motor cortex.

Figure 3 shows the network meta-analysis diagrams of eligible comparisons, where the blue circles represent the different interventions. The circle size represents the sample size. The straight line between the two circles represents a direct comparison between the two different interventions. The thicker the solid line, the greater the number of studies in that pairwise comparison.

#### 3.5.1 FMA-LE

A total of 29 included studies evaluated FMA-LE, involving 6 intervention protocols: HFrTMS-M1, LFrTMS-M1, iTBS-Cerebellum, iTBS-M1, dTMS-M1, and Placebo. A total of 1,685 patients were included. The inconsistency model evaluated global inconsistency, which showed *p* = 0.0772 (>0.05; Supplementary Figure 8A). The inconsistency test was not significant, so we used the consistency model. The node-splitting approach was used to evaluate local inconsistency. The test of local inconsistency from the node-splitting model showed a small percentage of inconsistency (1 of 6 comparisons), as detailed in Supplementary Table 2. 1 closed loop was formed for the 3 interventions, so we assessed the inconsistency of the closed loop. The results showed that the 95% CI included 0, and IF was close to 0, indicating that our NMA was highly credible (Supplementary Figure 9A).

**Figure 4:**
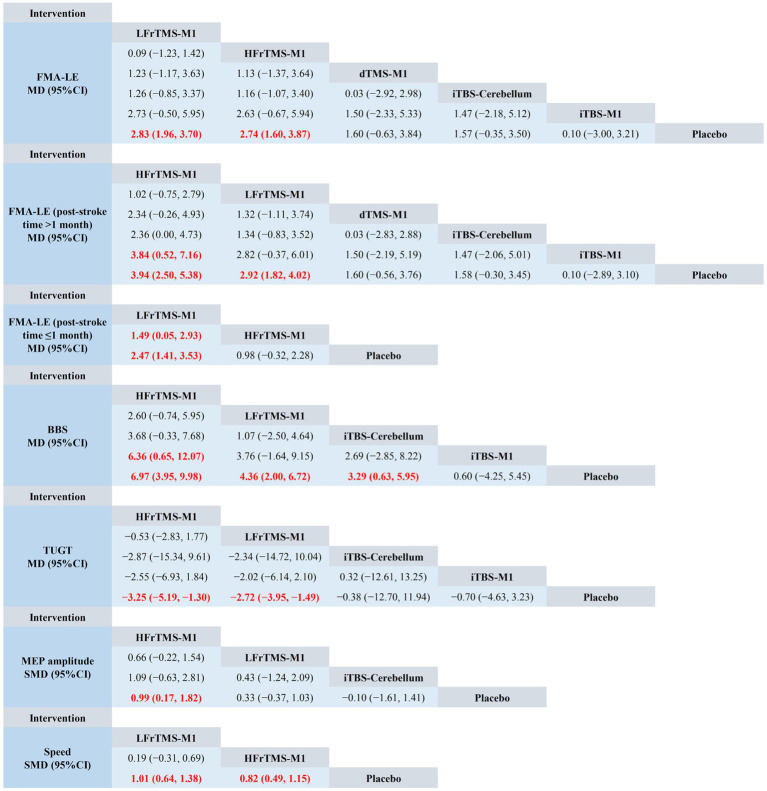
Network meta-analysis of head-to-head comparisons. Red and bold numbers are statistically significant. HFrTMS, high-frequency repetitive transcranial magnetic stimulation; LFrTMS, low-frequency repetitive transcranial magnetic stimulation; iTBS, intermittent theta-burst stimulation; dTMS, deep transcranial magnetic stimulation; M1, primary motor cortex.

**Figure 5:**
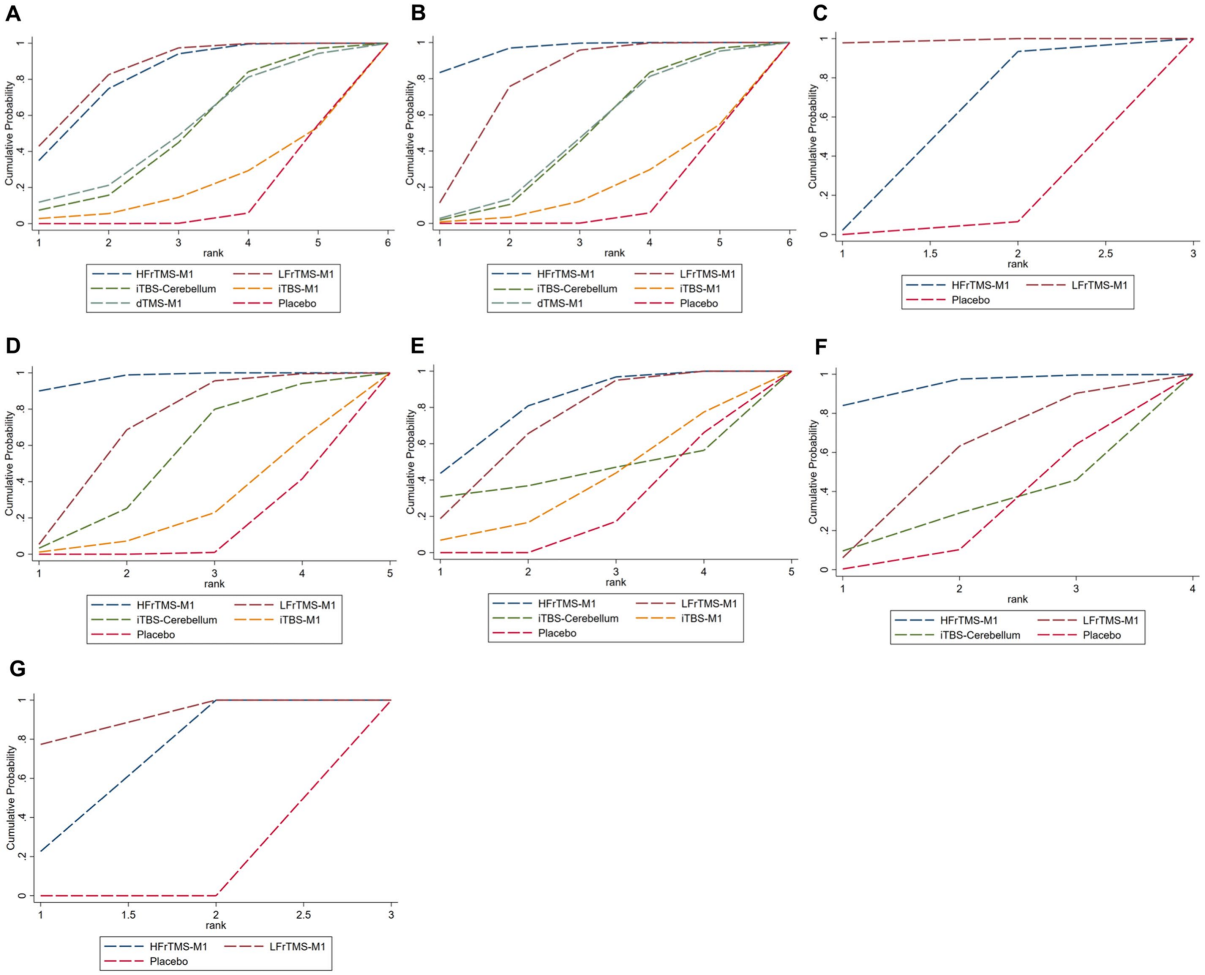
Cumulative probability ranking curve of different interventions. The vertical axis represents cumulative probabilities, while the horizontal axis represents ranks. **(A)** FMA-LE; **(B)** FMA-LE (post-stroke time > 1 month); **(C)** FMA-LE (post-stroke time ≤ 1 month); **(D)** BBS; **(E)** TUGT; **(F)** MEP amplitude; **(G)** Speed. HFrTMS, high-frequency repetitive transcranial magnetic stimulation; LFrTMS, low-frequency repetitive transcranial magnetic stimulation; iTBS, intermittent theta-burst stimulation; dTMS, deep transcranial magnetic stimulation; M1, primary motor cortex.

The NMA results showed that FMA-LE generated a total of 15 pairwise comparisons. Compared with Placebo, LFrTMS-M1 (MD = 2.83, 95% CI: 1.96 to 3.70) and HFrTMS-M1 (MD = 2.74, 95% CI: 1.60 to 3.87) significantly improved FMA-LE in stroke patients. There was no statistically significant difference between the other two interventions (*p* > 0.05; Figure 4). Figure 5A and Table 3 show the SUCRA rankings for all interventions. According to the analysis, LFrTMS-M1 (SUCRA, 84.6%) may be the most effective intervention to improve FMA-LE in stroke patients.

**Table 3:** SUCRA values of different interventions for outcomes.

Outcomes	HFrTMS-M1	LFrTMS-M1	iTBS-cerebellum	iTBS-M1	dTMS-M1	Placebo
FMA-LE	80.7%^b^	84.6%^a^	49.9%	21.2%	51.5%	12.2%
FMA-LE (post-stroke time > 1 month)	95.8%^a^	76.8%^b^	47.4%	20.0%	28.0%	11.9%
FMA-LE (post-stroke time ≤ 1 month)	47.8%^b^	98.9%^a^	NR	NR	NR	3.3%
BBS	96.8%^a^	67.7%^b^	51.8%	22.7%	NR	11.0%
TUGT	80.3%^a^	69.8%^b^	42.8%	36.2%	NR	20.8%
MEP amplitude	93.7%^a^	53.2%^b^	28.2%	NR	NR	24.9%
Speed	63.1%^b^	88.7%^a^	NR	NR	NR	0.0%

^a^Presents the first-ranking. ^b^Presents the second-ranking.

HFrTMS, high-frequency repetitive transcranial magnetic stimulation; LFrTMS, low-frequency repetitive transcranial magnetic stimulation, iTBS, intermittent theta-burst stimulation; dTMS, deep transcranial magnetic stimulation; M1, primary motor cortex; NR, not reported.

Different phases of stroke may lead to different therapeutic effects of rTMS. Therefore, we performed a subgroup analysis for FMA-LE according to the phase of stroke, including post-stroke time > 1 month and post-stroke time ≤ 1 month (Brunelli et al., 2019). The global consistency model of FMA-LE (post-stroke time > 1 month) and FMA-LE (post-stroke time ≤ 1 month) showed that the inconsistency test was not significant (Supplementary Figures 8B,C). There was a small percentage of inconsistency (1 of 3 comparisons) only for the local inconsistency test of FMA-LE (post-stroke time ≤ 1 month), details of which are provided in Supplementary Tables 3, 4.

For post-stroke time > 1 month, Figure 4 demonstrated that HFrTMS-M1 significantly improved FMA-LE (post-stroke time > 1 month) compared to iTBS-M1 (MD = 3.84, 95% CI: 0.52 to 7.16) and Placebo (MD = 3.94, 95% CI: 2.50 to 5.38). Figure 5B and Table 3 showed that the HFrTMS-M1 (SUCRA, 95.8%) was the best protocol. For post-stroke time ≤ 1 month, Figure 4 demonstrated that compared with the placebo, the LFrTMS-M1 (MD = 1.49, 95% CI: 0.05 to 2.93) and HFrTMS-M1 (MD = 2.47, 95% CI: 1.41 to 3.53) had better curative effects, with a statistically significant difference (*p* < 0.05). Figure 5C and Table 3 showed that the LFrTMS-M1 (SUCRA, 98.9%) was the best protocol. We found no publication bias by the funnel plot of publication bias and Egger’s test (Supplementary Figures 10B,C).

#### 3.5.2 BBS

A total of 11 included studies evaluated BBS, involving 5 intervention protocols: HFrTMS-M1, LFrTMS-M1, iTBS-Cerebellum, iTBS-M1, and Placebo. A total of 820 patients were included. The inconsistency model evaluated global inconsistency, which showed *p* = 0.2800 (>0.05; Supplementary Figure 8D). The inconsistency test was not significant, so we used the consistency model. The node-splitting approach was used to evaluate local inconsistency. The measured *p*-values were all greater than 0.05, indicating good local consistency (Supplementary Table 5). 1 closed loop was formed for the 3 interventions, so we assessed the inconsistency of the closed loop. The results showed that the 95% CI included 0, and IF was close to 0, indicating that our NMA was highly credible (Supplementary Figure 9D).

The NMA results showed that BBS generated a total of 10 pairwise comparisons. Compared with Placebo, HFrTMS-M1 (MD = 6.97, 95% CI: 3.95 to 9.98), LFrTMS-M1 (MD = 4.36, 95% CI: 2.00 to 6.72), and iTBS-Cerebellum (MD = 3.29, 95% CI: 0.63 to 5.95) significantly improved BBS in stroke patients. In addition, HFrTMS-M1 (MD = 6.36, 95% CI: 0.65 to 12.07) was significantly better than iTBS-M1 in improving BBS. Other pairwise comparisons showed no statistically significant differences (*p* > 0.05; Figure 4). Figure 5D and Table 3 show the SUCRA rankings for all interventions. According to the results of SUCRA analysis, HFrTMS-M1 (SUCRA, 96.8%) may be the most effective intervention to improve BBS in stroke patients.

#### 3.5.3 TUGT

A total of 8 included studies evaluated TUGT, involving 5 intervention protocols: HFrTMS-M1, LFrTMS-M1, iTBS-Cerebellum, iTBS-M1, and Placebo. A total of 382 patients were included. There was no closed loop, so we did not need to perform a consistency check. The NMA results showed that TUGT generated a total of 10 pairwise comparisons.

Compared with Placebo, HFrTMS-M1 (MD = −3.25, 95% CI: −5.19 to −1.30) and LFrTMS-M1 (MD = −2.72, 95% CI: −3.95 to −1.49) significantly improved TUGT in stroke patients. There was no statistically significant difference between the other two interventions (*p* > 0.05; Figure 4). Figure 5E and Table 3 show the SUCRA rankings for all interventions. According to the analysis, HFrTMS-M1 (SUCRA, 80.3%) may be the most effective intervention to improve TUGT in stroke patients.

#### 3.5.4 MEP amplitude

A total of 8 included studies evaluated MEP amplitude, involving 4 intervention protocols: HFrTMS-M1, LFrTMS-M1, iTBS-Cerebellum, and Placebo. A total of 246 patients were included. The inconsistency model evaluated global inconsistency, which showed *p* = 0.5656 (>0.05; Supplementary Figure 8E). The inconsistency test was not significant, so we used the consistency model. The node-splitting approach was used to evaluate local inconsistency. The measured *p*-values were all greater than 0.05, indicating good local consistency (Supplementary Table 3). 1 closed loop was formed for the 3 interventions, so we assessed the inconsistency of the closed loop. The results showed that the 95% CI included 0, and IF was close to 0, indicating that our NMA was highly credible (Supplementary Figure 9E).

The NMA results showed that MEP amplitude generated a total of 6 pairwise comparisons. Compared with Placebo, HFrTMS-M1 (SMD = 0.99, 95% CI: 0.17 to 1.82) significantly improved MEP amplitude in stroke patients. There was no statistically significant difference between the other two interventions (*p* > 0.05; Figure 4). Figure 5F and Table 3 show the SUCRA rankings for all interventions. According to the analysis, HFrTMS-M1 (SUCRA, 93.7%) may be the most effective intervention to improve MEP amplitude in stroke patients.

#### 3.5.5 Speed

A total of 13 included studies evaluated speed, involving 3 intervention protocols: HFrTMS-M1, LFrTMS-M1, and Placebo. A total of 667 patients were included. There was no closed loop, so we did not need to perform a consistency check. The NMA results showed that speed generated a total of 3 pairwise comparisons.

Compared with Placebo, LFrTMS-M1 (SMD = 1.01, 95% CI: 0.64 to 1.38) and HFrTMS-M1 (SMD = 0.82, 95% CI: 0.49 to 1.15) significantly improved speed in stroke patients. There was no statistically significant difference between the other two interventions (*p* > 0.05; Figure 4). Figure 5G and Table 3 show the SUCRA rankings for all interventions. According to the analysis, LFrTMS-M1 (SUCRA, 88.7%) may be the most effective intervention to improve speed in stroke patients.

### 3.6 Publication bias

**Figure 6:**
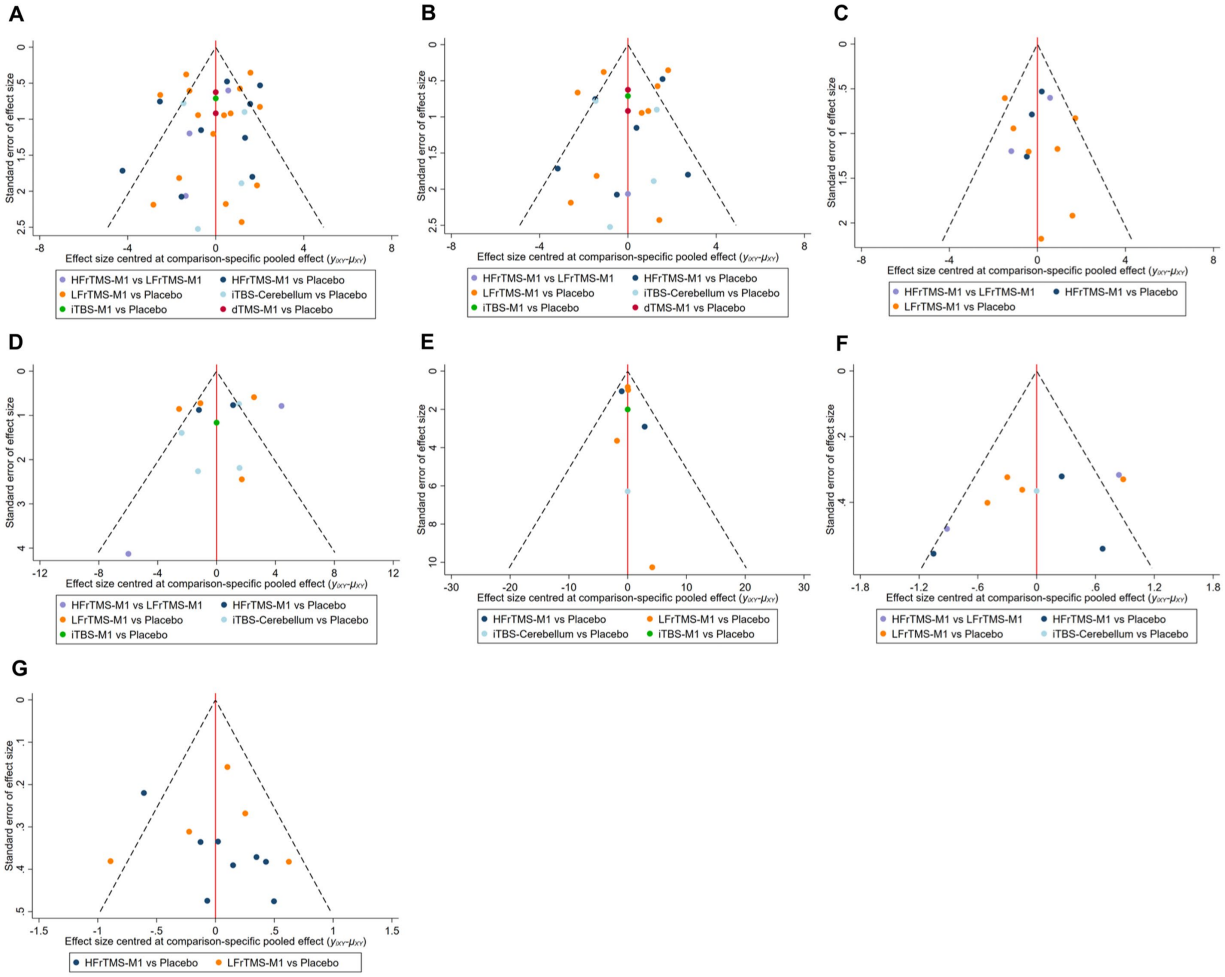
Funnel plot of publication bias. **(A)** FMA-LE; **(B)** FMA-LE (post-stroke time > 1 month); **(C)** FMA-LE (post-stroke time ≤ 1 month); **(D)** BBS; **(E)** TUGT; **(F)** MEP amplitude; **(G)** Speed. HFrTMS, high-frequency repetitive transcranial magnetic stimulation; LFrTMS, low-frequency repetitive transcranial magnetic stimulation; iTBS, intermittent theta-burst stimulation; dTMS, deep transcranial magnetic stimulation; M1, primary motor cortex.

This study evaluated publication bias for FMA-LE, FMA-LE (post-stroke time > 1 month), FMA-LE (post-stroke time ≤ 1 month), BBS, TUGT, MEP amplitude, and speed using the funnel plot of publication bias (Figure 6) and Egger’s test. The findings revealed that most points were evenly distributed along both sides of the midline and were primarily focused there, indicating that our results were robust and there was no significant publication bias. In addition, we used Egger’s test for secondary validation of publication bias. The results showed FMA-LE (Egger’s test *p* = 0.273; Supplementary Figure 10A), FMA-LE (post-stroke time > 1 month; Egger’s test *p* = 0.807; Supplementary Figure 10B), FMA-LE (post-stroke time ≤ 1 month; Egger’s test *p* = 0.601; Supplementary Figure 10C), BBS (Egger’s test *p* = 0.843; Supplementary Figure 10D), TUGT (Egger’s test *p* = 0.123; Supplementary Figure 10E), MEP amplitude (Egger’s test *p* = 0.089; Supplementary Figure 10F), and speed (Egger’s test *p* = 0.556; Supplementary Figure 10G), indicating that there was no publication bias in this study.

### 3.7 Adverse events

Among the 38 included studies, 16 had no adverse events during the course of the experiment, 7 reported adverse events in detail, and the remaining studies did not describe adverse events (Supplementary Table 7). The adverse events reported were mild, such as headache, dizziness, nausea, and vomiting.

## 4 Discussion

Stroke is a common disease worldwide and causes severe disabilities for patients. More than two-thirds of stroke survivors have post-stroke sequelae, including impairment in motor function, balance, gait, and ADL (Paul et al., 2007). Improving lower extremity motor function and balance ability can significantly impact gait function, ADL, and quality of life in stroke patients (Smith et al., 2017). Although the use of rTMS for stroke has attracted considerable attention, there is still a lack of consensus on the optimal protocol for rTMS to improve lower extremity motor function in stroke patients. To the best of our knowledge, this is the first NMA to compare the effects of different rTMS protocols on lower extremity motor function in stroke patients by taking stimulation frequency, stimulation target, and stimulation mode of rTMS and post-stroke time into account simultaneously.

The FMA-LE can predict lower extremity motor recovery in individuals with stroke (Balasubramanian et al., 2016). This assessment exhibits good internal consistency and reliability, discriminative validity, and responsiveness to interventions (Hsieh et al., 2009). This study found that compared with Placebo, LFrTMS-M1 and HFrTMS-M1 significantly improved FMA-LE in stroke patients, and LFrTMS in the contralateral hemisphere was more effective than HFrTMS in the ipsilateral hemisphere. Xie et al. (2021a) suggested that LFrTMS in the contralateral hemisphere was more effective than HFrTMS in the ipsilateral hemisphere, which was consistent with our findings. However, a novel finding from this NMA is that the subgroup analyses of FMA-LE showed that at ≤1 month after stroke, HFrTMS-M1 was the optimal stimulation protocol for improving stroke patients’ lower extremity motor function. At ≤1 month after stroke, LFrTMS-M1 was the best protocol, which was superior to HFrTMS-M1. The difference between these two studies was that when grouping rTMS protocols, we considered that the same rTMS mode applied to different stimulation targets would produce different therapeutic effects. Therefore, we divided stimulation targets into M1 and Cerebellum rather than simply grouping very different stimulation targets together, which allowed the intervention protocol to be more refined. Second, we included more articles that met our research objectives to expand the sample size. Finally, we also carried out a subgroup analysis of the patients on the efficacy of rTMS at different protocols to improve the accuracy of the outcome evidence. In addition, we also found that 10 Hz and 1 Hz were the most commonly used stimulation frequencies for HFrTM and LFrTMS, respectively, regarding rTMS prescription settings, which was the same conclusion as Fan et al. (2021). Meanwhile, rTMS sessions of 15 or 20 min each and lasting 3 or 4 weeks were the most common. In clinical practice, clinicians or rehabilitation therapists can flexibly formulate the best stimulation prescription according to the specific situation of patients and the recommended protocols mentioned above.

In fact, early hyperexcitability and increased interhemispheric inhibition of the contralesional motor cortex have been demonstrated using TMS after unilateral stroke. Therefore, LFrTMS can effectively improve the motor function of stroke patients by reducing the excitability of the motor cortex of the contralateral hemisphere to restore the balance of competitive inhibition between the two hemispheres in the acute phase of stroke. However, in the post-stroke convalescent phase, the interhemispheric competition is less pronounced than in the acute phase, as it is commonly observed that the transcallosal asymmetry decreases with time (Swayne et al., 2008). LFrTMS may reduce the compensatory effect of the contralateral hemisphere by inhibiting its excitability, thereby hindering functional recovery after stroke. Therefore, HFrTMS-M1 may be more effective than LFrTMS-M1 in the convalescent phase of stroke. Xia et al. (2022) also recommended the application of HFrTMS in patients with stroke patients during the convalescent phase.

The BBS is the most widely used clinical scale for assessing balance performance in individuals with neurological conditions, including static and dynamic balance (Neuls et al., 2011). The sum of the scores for the 14 items (each item was rated from 0 to 4) yielded a balance score ranging from 0 to 56 (Neuls et al., 2011). TUGT is a rapid and quantitative assessment of dynamic balance and functional walking ability and is closely related to other measures of gait and balance in stroke patients (Flansbjer et al., 2005). TUGT assesses the time taken to complete a series of actions, including standing up from a chair, walking forward three meters, turning, and returning to the chair. According to the ranking probability of our NMA, HFrTMS-M1 was more advantageous in improving BBS and TUGT. Therefore, we recommend HFrTMS-M1 as a complementary rehabilitation therapy to improve balance function in stroke patients in clinical practice.

Walking speed can reflect the recovery of lower extremity function and walking quality in stroke patients (Patterson et al., 2008). Our NMA results showed that compared with Placebo, LFrTMS-M1 and HFrTMS-M1 significantly improved the speed in stroke patients. LFrTMS-M1 had more advantages in improving the speed of stroke patients. However, Tung et al. (2019) found that HFrTMS was superior to LFrTMS in improving speed in stroke patients. Further understanding of the relationship between different rTMS protocols and walking speed is needed in the future. Transcranial magnetic stimulation (TMS) is a non-invasive neuromodulation technique that produces pulsed magnetic fields that form induced currents in the motor cortex of the brain. After the induced current stimulates one side of the motor cortex, the conduction nerve impulses are transmitted downward, which will cause the target muscle on the opposite side of the subject to produce action potentials, called motor-evoked potentials (MEPs). MEPs is a quantitative evaluation index of central motor conduction function, which can objectively reflect the excitability of the motor cortex. In this study, we used MEP amplitude to assess the functional status of motor conduction pathways, and the results showed that HFrTMS-M1 was the most effective in improving MEP amplitude. However, MEP amplitude included only a few studies, meaning this ranking result should be treated critically. In addition, to ensure the objectivity of the study results, more high-quality RCTs with large sample sizes are needed for further verification.

Like TMS, functional magnetic resonance imaging (fMRI), electroencephalography (EEG), and other neuroimaging techniques are also of great significance in the assessment of motor function in stroke. fMRI can evaluate the neurovascular response induced by rTMS, and the activity changes of brain nerves can be observed through intuitive and visual images (Bergmann et al., 2016). In addition, fMRI can also be used to study the excitability and functional connectivity of the cerebral cortex and subcortex in stroke patients under rTMS intervention. Guo et al. (2021) used fMRI to find that both LFrTMS stimulation in the unaffected hemisphere and HFrTMS stimulation in the affected hemisphere could promote the reorganization of the motor network, and the changes in functional connectivity between the contralateral PMA and the ipsilateral M1, and between the bilateral M1 induced by rTMS were related to motor recovery. Transcranial magnetic stimulation combined with electroencephalography (TMS-EEG) is a new evaluation method in recent years. TMS-EEG can reflect the direct relationship between brain regions and motor function in stroke patients and predict the recovery of motor function after stroke. Koch et al. (2019) performed motor assessment on 34 stroke subjects who received iTBS-Cerebellum or sham iTBS treatment by Fugl-Meyer scale, gait analysis, and so on, and recorded cerebral cortical activity through TMS-EEG to achieve the combination of treatment and assessment feedback. At present, there are relatively few studies using fMRI and EEG to explore the treatment of rTMS to promote the recovery of dysfunction after stroke, and most of the trials are small in sample size and scale. In the future, large-sample, multi-center, and high-quality RCTs should be carried out, and the results of imaging and electrophysiology of stroke patients should be comprehensively analyzed to improve the accuracy and scientificity of rehabilitation efficacy evaluation.

The M1 is the most essential part of the motor cortex in the human cerebral cortex, located in the precentral gyrus. M1 is the most frequently stimulated target in noninvasive brain stimulation studies for post-stroke gait and balance recovery (Parikh et al., 2023). Among the 38 studies included in this article, 33 studies selected M1 as the stimulation target. Recent electrophysiological and imaging evidence underlined that a large motor network includes other key brain areas during the process of post-stroke functional recovery (Koch and Hummel, 2017). The cerebellum is a crucial structure involved in balance and motor control, and it is essential in motor adaptation and learning processes. Therefore, the cerebellum has been proposed as one of the alternative targets of M1. A total of 5 studies in our NMA used the cerebellum as the stimulation target and selected the iTBS mode for intervention. iTBS is a new rTMS mode that lasts only about 5 min, with the characteristics of short time-consuming, low intensity, and strong effect. iTBS can induce long-term potentiation, which helps promote neural plasticity and produce a safer and lasting intervention effect for stroke patients. Our findings showed that iTBS-Cerebellum can significantly improve balance function in stroke patients. Liao et al. (2024) compared the efficacy and safety of iTBS to the cerebellum or M1 on balance and motor recovery in stroke patients. They found that both iTBS-M1 and iTBS-Cerebellum could improve balance function and that iTBS-Cerebellum, but not iTBS-M1, had a more significant effect on motor recovery. Like our findings, Manto et al. (2012) suggested that iTBS-Cerebellum could be a potential therapeutic approach to improve balance and gait function in stroke patients. Thus, iTBS-Cerebellum may be a valuable new therapeutic option in stroke rehabilitation programs. At the same time, our NMA also included a study on the iTBS-M1 protocol, but the limited number of studies and participants may have led to inaccurate results.

It is worth noting that among the included studies, Chieffo et al. (2014) and Chieffo et al. (2021) used H-coil. The H-coil differs from conventional figure-of-8 coil and circular coil in that it can stimulate deeper cortical areas and neural networks (Roth et al., 2014). Roth et al. (2007) found that H-coil, figure-of-8 coil, and double-cone coil could generate the maximum induced electric field in the surface region of the saline head model at 0.9% concentration. With the increased distance from the simulated skull to the brain tissue, the induced electric field generated by the figure-of-8 coil and the double-cone coil decreased to less than 10% of the maximum induced electric field at 6 cm. In comparison, the electric field intensity of the H-coil was more than 63% of the maximum induced electric field. H-coil can theoretically stimulate deeper leg-related cortical motor areas within the intercerebral fissure approximately 3 to 4 cm below the skull. However, the results of Chieffo et al. (2014) and Chieffo et al. (2021) did not show a favorable advantage of dTMS. We suspect this may be due to the small number of current studies. In the future, more RCTs are needed to confirm the applicability and safety of dTMS in stroke patients.

## 5 Limitations

The study also has some limitations, including: (1) Coil type, stimulation intensity, total number of pulses, and duration of intervention were not exactly the same among the included studies, resulting in potential heterogeneity. (2) Despite including the full stimulation protocol in this analysis, the iTBS-M1 and dTMS-M1 groups accounted for 2.6 and 5.3% of the total data, respectively. This affects, to some extent, the quality of the conclusions of this study. (3) The age and disease severity of the included patients were slightly different, and some data indicators will be affected. Further subgroup analysis according to age and disease severity is needed in the future. (4) Adverse events may not be strictly reported in the included studies, so the safety of each intervention protocol needs to be further studied.

## 6 Conclusion

In this NMA, we found differences in the therapeutic effects between different rTMS protocols. Considering the impact of the stroke phase on the lower extremity motor function, the current research evidence shows that HFrTMS-M1 may be the preferred stimulation protocol to improve the lower extremity motor function of patients for post-stroke time > 1 month, and LFrTMS-M1 for post-stroke time ≤ 1 month. In the future, high-quality, large-sample, multi-center, and long-term follow-up RCTs are needed to verify the conclusions of this study.

## Data availability statement

The original contributions presented in the study are included in the article/Supplementary material, further inquiries can be directed to the corresponding authors.

## Author contributions

CWa: Software, Writing – original draft. QZ: Software, Writing – original draft. LZ: Data curation, Methodology, Supervision, Writing – review & editing. DZ: Data curation, Methodology, Supervision, Writing – review & editing. YX: Data curation, Methodology, Supervision, Writing – review & editing. ZL: Data curation, Methodology, Supervision, Writing – review & editing. CWu: Supervision, Writing – review & editing, Data curation, Methodology. SW: Supervision, Writing – review & editing, Data curation, Methodology. MY: Supervision, Writing – review & editing. LW: Supervision, Writing – review & editing.
