# Supplementary material for: Comparative efficacy of different repetitive transcranial magnetic stimulation protocols for lower extremity motor function in stroke patients: a network meta-analysis
Source: Front Neurosci. 2024 Feb 15;18:1352212. doi: 10.3389/fnins.2024.1352212 (PMC10902063; doi:10.3389/fnins.2024.1352212)
Supplement: Supplementary file 1 [file Data_Sheet_1.docx]

Supplementary Material

## 1. Supplementary Figures


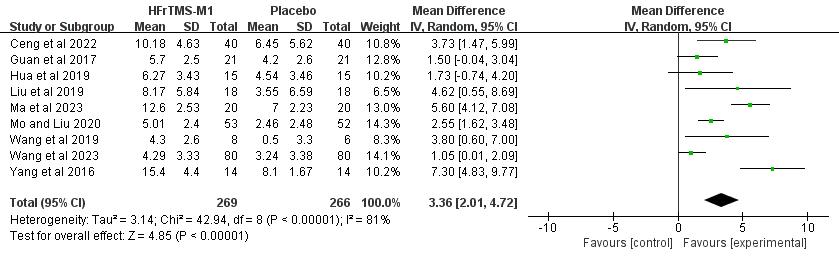

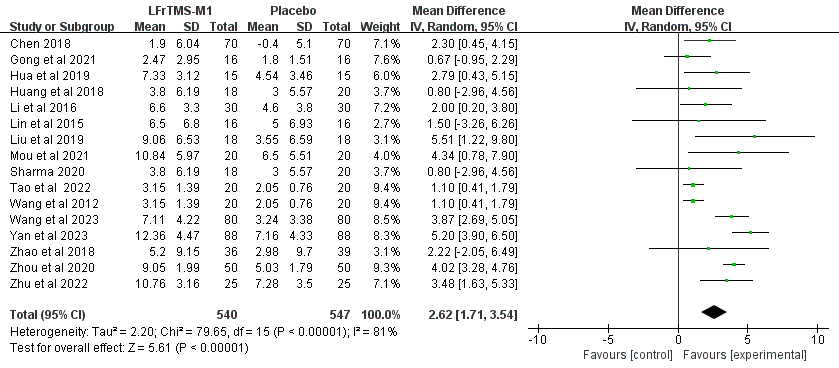

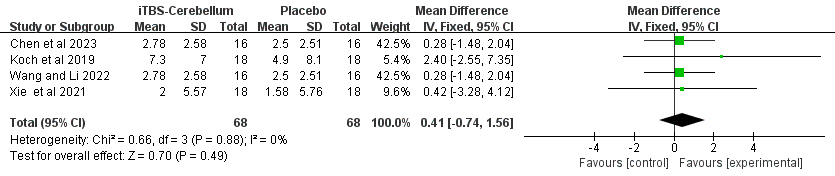

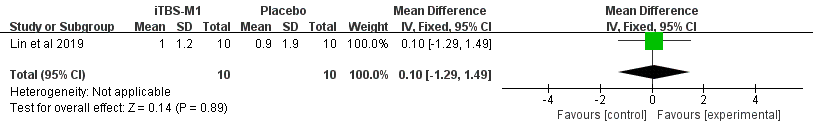

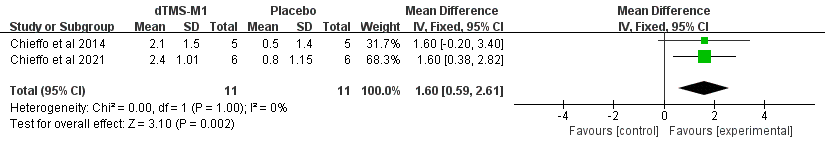

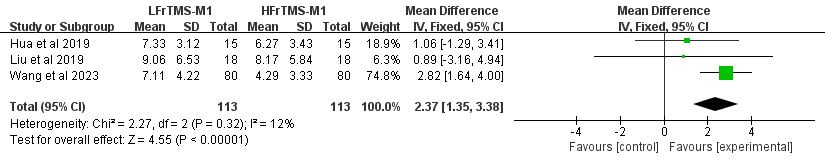


**Supplementary Figure 1.** Forest plots of the pairwise meta-analysis for FMA-LE. HFrTMS, high-frequency repetitive transcranial magnetic stimulation; LFrTMS, low-frequency repetitive transcranial magnetic stimulation; iTBS, intermittent theta-burst stimulation; dTMS, deep transcranial magnetic stimulation; M1, primary motor cortex.


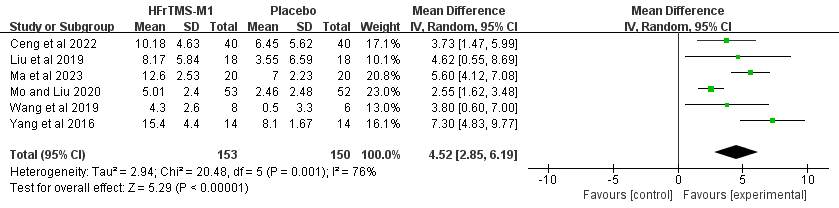

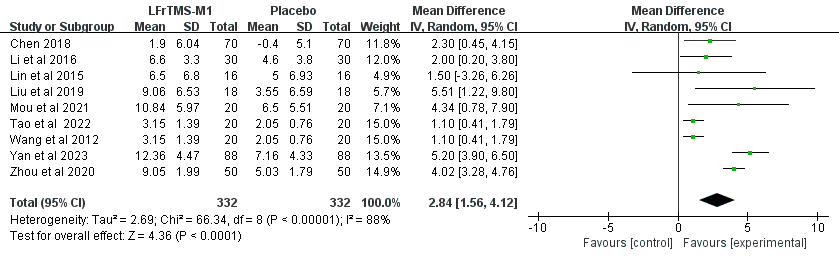

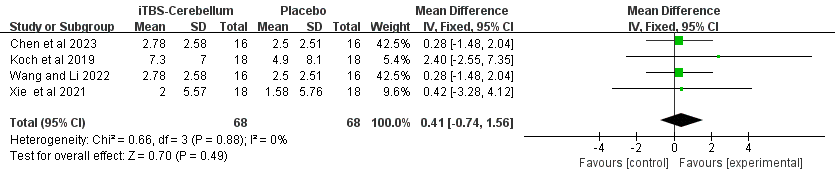

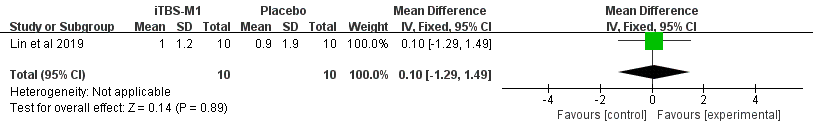

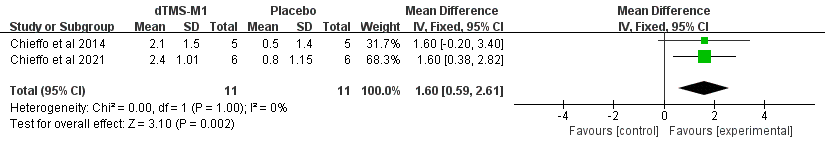

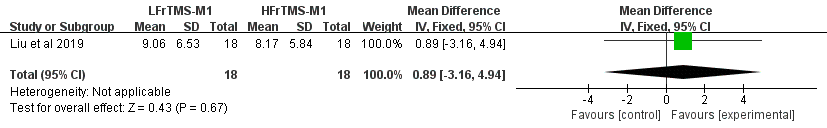


**Supplementary Figure 2.** Forest plots of the pairwise meta-analysis for FMA-LE (post-stroke time >1 month). HFrTMS, high-frequency repetitive transcranial magnetic stimulation; LFrTMS, low-frequency repetitive transcranial magnetic stimulation; iTBS, intermittent theta-burst stimulation; dTMS, deep transcranial magnetic stimulation; M1, primary motor cortex.


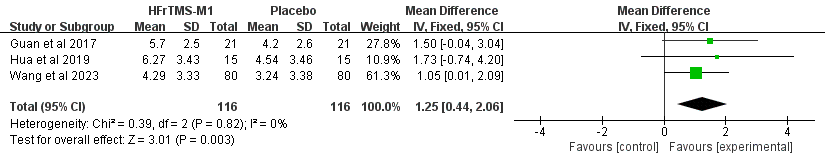

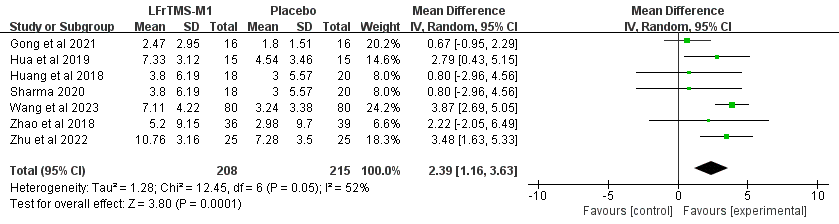

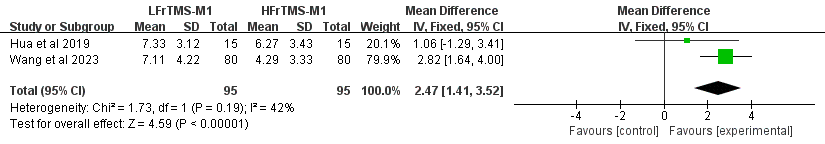


**Supplementary Figure 3.** Forest plots of the pairwise meta-analysis for FMA-LE (post-stroke time ≤1 month). HFrTMS, high-frequency repetitive transcranial magnetic stimulation; LFrTMS, low-frequency repetitive transcranial magnetic stimulation; M1, primary motor cortex.


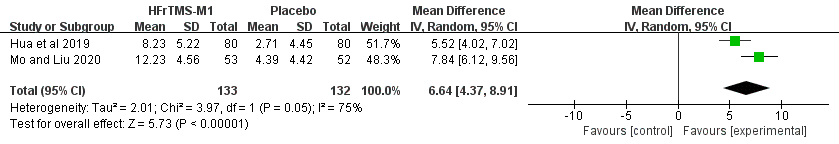

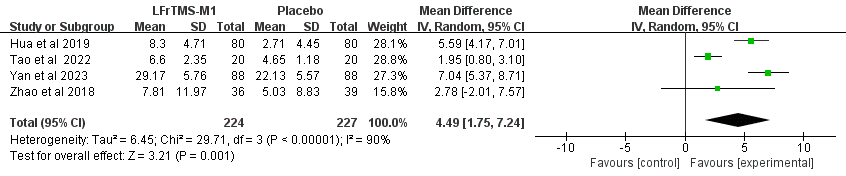

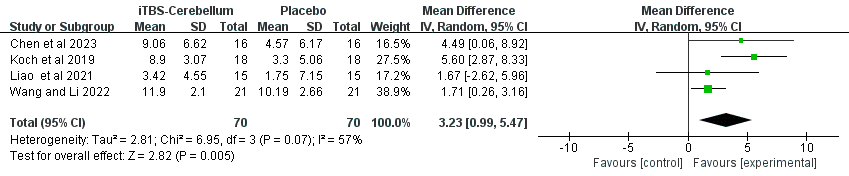

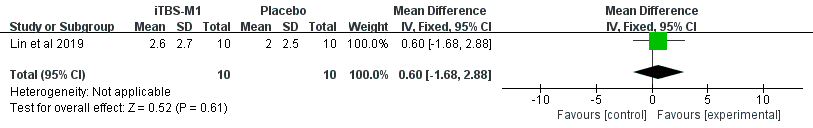

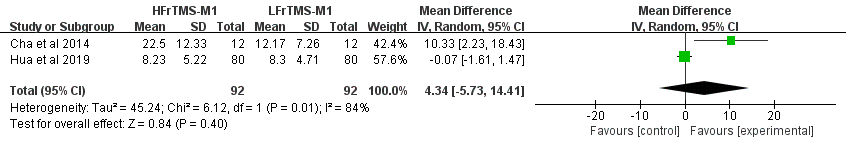


**Supplementary Figure 4.** Forest plots of the pairwise meta-analysis for BBS. HFrTMS, high-frequency repetitive transcranial magnetic stimulation; LFrTMS, low-frequency repetitive transcranial magnetic stimulation; iTBS, intermittent theta-burst stimulation; M1, primary motor cortex.


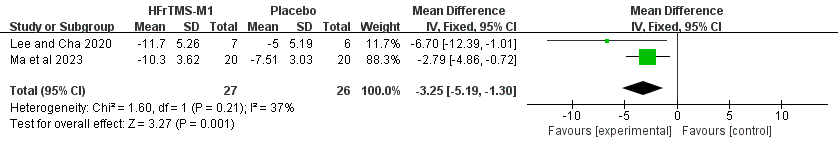

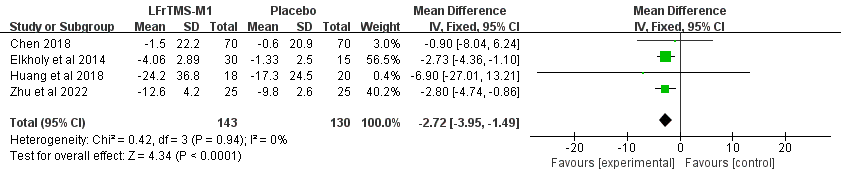

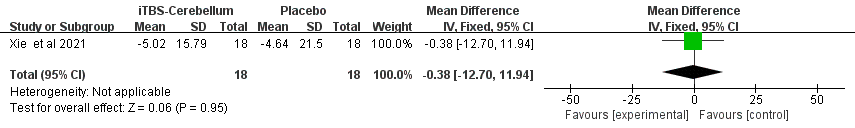

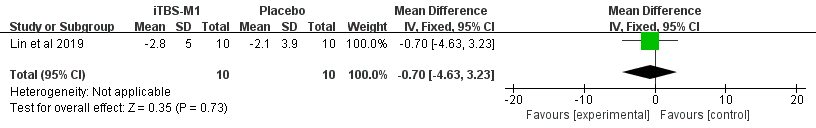


**Supplementary Figure 5.** Forest plots of the pairwise meta-analysis for TUGT. HFrTMS, high-frequency repetitive transcranial magnetic stimulation; LFrTMS, low-frequency repetitive transcranial magnetic stimulation; iTBS, intermittent theta-burst stimulation; M1, primary motor cortex.

**
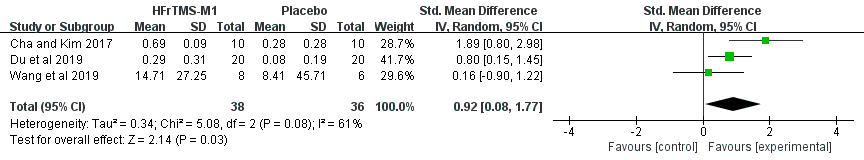

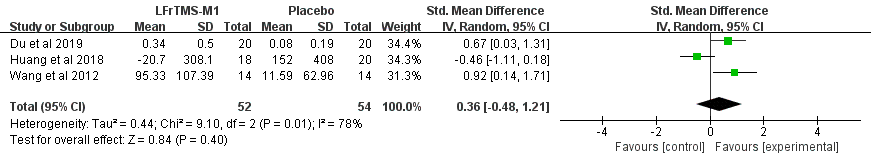

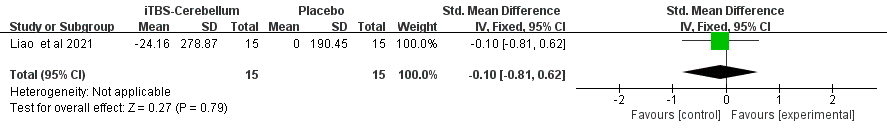

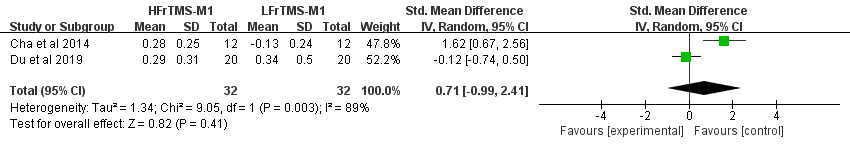
**

**Supplementary Figure 6.** Forest plots of the pairwise meta-analysis for MEP amplitude. HFrTMS, high-frequency repetitive transcranial magnetic stimulation; LFrTMS, low-frequency repetitive transcranial magnetic stimulation; iTBS, intermittent theta-burst stimulation; M1, primary motor cortex.


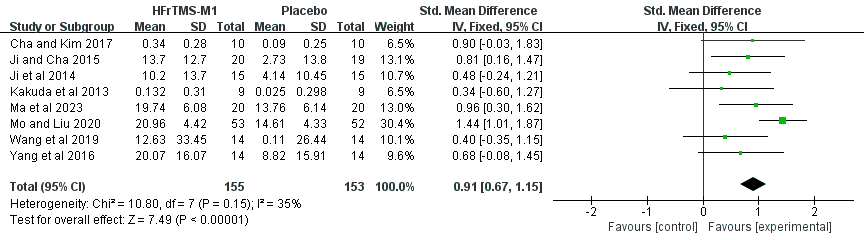

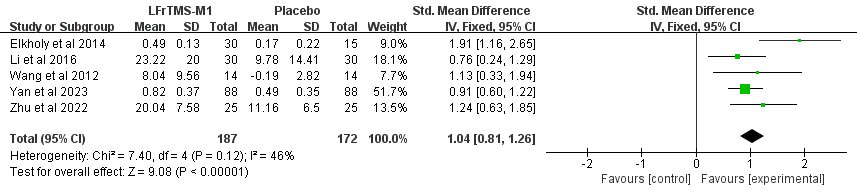


**Supplementary Figure 7.** Forest plots of the pairwise meta-analysis for speed. HFrTMS, high-frequency repetitive transcranial magnetic stimulation; LFrTMS, low-frequency repetitive transcranial magnetic stimulation; M1, primary motor cortex.


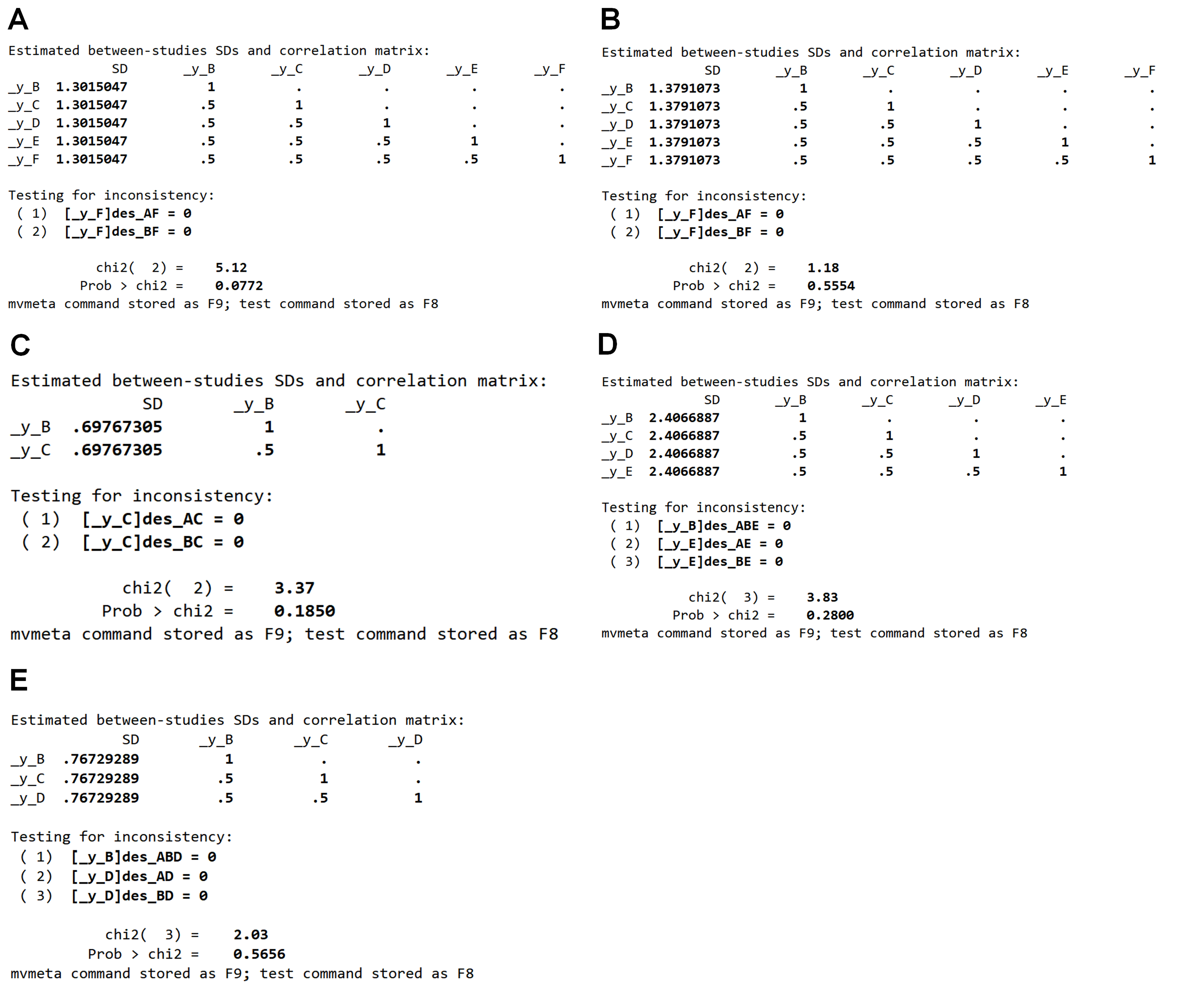


## Supplementary Figure 8. Global consistency test. (A) FMA-LE; (B) FMA-LE (post-stroke time >1 month); (C) FMA-LE (post-stroke time ≤1 month); (D) BBS; (E) MEP amplitude.

##



## Supplementary Figure 9. Loop consistency test. (A) FMA-LE; (B) FMA-LE (post-stroke time >1 month); (C) FMA-LE (post-stroke time ≤1 month); (D) BBS; (E) MEP amplitude.





## Supplementary Figure 10. Egger’s test. (A) FMA-LE; (B) FMA-LE (post-stroke time >1 month); (C) FMA-LE (post-stroke time ≤1 month); (D) BBS; (E) TUGT; (F) MEP amplitude; (G) Speed.

**2. Supplementary Tables**

**Supplementary Table 1.** Search strategy (using PubMed as an example).

| Search | Query |
| --- | --- |
| #1 | (((“Transcranial Magnetic Stimulation”[Mesh]) OR (((((((((((((((((Magnetic Stimulation, Transcranial[Title/Abstract]) OR (Magnetic Stimulations, Transcranial[Title/Abstract])) OR (Stimulation, Transcranial Magnetic[Title/Abstract])) OR (Stimulations, Transcranial Magnetic[Title/Abstract])) OR (Transcranial Magnetic Stimulations[Title/Abstract])) OR (Transcranial Magnetic Stimulation, Single Pulse[Title/Abstract])) OR (Transcranial Magnetic Stimulation, Paired Pulse[Title/Abstract])) OR (Transcranial Magnetic Stimulation, Repetitive[Title/Abstract])) OR (repetitive transcranial magnetic stimulation[Title/Abstract])) OR (TMS[Title/Abstract])) OR (rTMS[Title/Abstract])) OR (magnetic stimulation[Title/Abstract])) OR (TBS[Title/Abstract])) OR (iTBS[Title/Abstract])) OR (cTBS[Title/Abstract])) OR (Theta burst stimulation[Title/Abstract])) OR (θ-brust stimulation[Title/Abstract]))) |
| #2 | ((“Stroke”[Mesh]) OR (((((((((((((((((((((((((((((((((Strokes[Title/Abstract]) OR (Cerebrovascular Accident[Title/Abstract])) OR (Cerebrovascular Accidents[Title/Abstract])) OR (CVA (Cerebrovascular Accident[Title/Abstract]))) OR (CVAs (Cerebrovascular Accident[Title/Abstract]))) OR (Cerebrovascular Apoplexy[Title/Abstract])) OR (Apoplexy, Cerebrovascular[Title/Abstract])) OR (Vascular Accident, Brain[Title/Abstract])) OR (Brain Vascular Accident[Title/Abstract])) OR (Brain Vascular Accidents[Title/Abstract])) OR (Vascular Accidents, Brain[Title/Abstract])) OR (Cerebrovascular Stroke[Title/Abstract])) OR (Cerebrovascular Strokes[Title/Abstract])) OR (Stroke, Cerebrovascular[Title/Abstract])) OR (Strokes, Cerebrovascular[Title/Abstract])) OR (Apoplexy[Title/Abstract])) OR (Cerebral Stroke[Title/Abstract])) OR (Cerebral Strokes[Title/Abstract])) OR (Stroke, Cerebral[Title/Abstract])) OR (Strokes, Cerebral[Title/Abstract])) OR (Stroke, Acute[Title/Abstract])) OR (Acute Stroke[Title/Abstract])) OR (Acute Strokes[Title/Abstract])) OR (Strokes, Acute[Title/Abstract])) OR (Cerebrovascular Accident, Acute[Title/Abstract])) OR (Acute Cerebrovascular Accident[Title/Abstract])) OR (Acute Cerebrovascular Accidents[Title/Abstract])) OR (Cerebrovascular Accidents, Acute[Title/Abstract])) OR (apoplexy[Title/Abstract])) OR (hemiplegia[Title/Abstract])) OR (cerebrovascular disease[Title/Abstract])) OR (cerebral infarction[Title/Abstract])) OR (cerebral hemorrhage[Title/Abstract]))) |
| #3 | (“randomized controlled trial”[Publication Type] OR “random”[Title/Abstract] OR “random allocation”[Title/Abstract] OR “single blind”[Title/Abstract] OR “double blind”[Title/Abstract] OR “RCT”[Title/Abstract] OR “controlled clinical trials”[Title/Abstract])) |
| #4 | #1 AND #2 AND #3 |

**Supplementary Table 2.** Local consistency test for FMA-LE.

| Side | Direct |  | Indirect |  | Difference |  |  | tau |
| --- | --- | --- | --- | --- | --- | --- | --- | --- |
|  | Coef. | Std. Err. | Coef. | Std. Err. | Coef. | Std. Err. | P>z |  |
| **HFrTMS-M1 vs LFrTMS-M1** | 1.901491 | .9986675 | -1.034572 | .8078265 | 2.936063 | 1.281284 | 0.022 | 1.267057 |
| **HFrTMS-M1 vs Placebo** | -3.011828 | .5831852 | .7433038 | 2.033698 | -3.755132 | 2.118202 | 0.076 | 1.351758 |
| **LFrTMS-M1 vs Placebo** | -2.677971 | .4274436 | -7.049607 | 2.215831 | 4.371636 | 2.25603 | 0.053 | 1.299355 |
| **iTBS-Cerebellum vs Placebo** | -1.570918 | .9823828 | -5.484529 | 105.4292 | 3.913611 | 105.4339 | 0.970 | 1.416277 |
| **iTBS-M1 vs Placebo** | -.1 | 1.584595 | -5.472782 | 98.07204 | 5.372782 | 98.08705 | 0.956 | 1.416313 |
| **dTMS-M1 vs Placebo** | -1.6 | 1.140811 | -5.473777 | 54.82391 | 3.873777 | 54.83593 | 0.944 | 1.416377 |

HFrTMS, high-frequency repetitive transcranial magnetic stimulation; LFrTMS, low-frequency repetitive transcranial magnetic stimulation; iTBS, intermittent theta-burst stimulation; dTMS, deep transcranial magnetic stimulation; M1, primary motor cortex.

**Supplementary Table 3.** Local consistency test for FMA-LE (post-stroke time >1 month).

| Side | Direct |  | Indirect |  | Difference |  |  | tau |
| --- | --- | --- | --- | --- | --- | --- | --- | --- |
|  | Coef. | Std. Err. | Coef. | Std. Err. | Coef. | Std. Err. | P>z |  |
| **HFrTMS-M1 vs LFrTMS-M1** | .812818 | 2.47537 | -1.309826 | .9802822 | 2.122644 | 2.66502 | 0.426 | 1.369145 |
| **HFrTMS-M1 vs Placebo** | -4.086699 | .7479735 | .6953834 | 4.364308 | -4.782082 | 4.437545 | 0.281 | 1.350461 |
| **LFrTMS-M1 vs Placebo** | -2.898963 | .5750109 | -4.38255 | 4.52564 | 1.483587 | 4.561841 | 0.745 | 1.382398 |
| **iTBS-Cerebellum vs Placebo** | -1.576085 | .9555814 | -7.895656 | 105.4402 | 6.319571 | 105.4446 | 0.952 | 1.353008 |
| **iTBS-M1 vs Placebo** | -.1000001 | 1.528317 | -7.883487 | 98.07091 | 7.783486 | 98.08503 | 0.937 | 1.353053 |
| **dTMS-M1 vs Placebo** | -1.6 | 1.10145 | -7.886278 | 54.8294 | 6.286278 | 54.84062 | 0.909 | 1.353144 |

HFrTMS, high-frequency repetitive transcranial magnetic stimulation; LFrTMS, low-frequency repetitive transcranial magnetic stimulation; iTBS, intermittent theta-burst stimulation; dTMS, deep transcranial magnetic stimulation; M1, primary motor cortex.

**Supplementary Table 4.** Local consistency test for FMA-LE (post-stroke time ≤1 month).

| Side | Direct |  | Indirect |  | Difference |  |  | tau |
| --- | --- | --- | --- | --- | --- | --- | --- | --- |
|  | Coef. | Std. Err. | Coef. | Std. Err. | Coef. | Std. Err. | P>z |  |
| **HFrTMS-M1 vs LFrTMS-M1** | 2.279084 | .7549535 | -.0547999 | 1.121996 | 2.333884 | 1.356643 | 0.085 | .6412764 |
| **HFrTMS-M1 vs Placebo** | -1.294569 | .526142 | 2.373658 | 1.669552 | -3.668227 | 1.731234 | 0.034 | .4725198 |
| **LFrTMS-M1 vs Placebo** | -2.400198 | .5754518 | -3.892022 | 3.001135 | 1.491823 | 3.049371 | 0.625 | .9933003 |

HFrTMS, high-frequency repetitive transcranial magnetic stimulation; LFrTMS, low-frequency repetitive transcranial magnetic stimulation; M1, primary motor cortex.

**Supplementary Table 5.** Local consistency test for BBS.

| Side | Direct |  | Indirect |  | Difference |  |  | tau |
| --- | --- | --- | --- | --- | --- | --- | --- | --- |
|  | Coef. | Std. Err. | Coef. | Std. Err. | Coef. | Std. Err. | P>z |  |
| **HFrTMS-M1 vs LFrTMS-M1** | -2.288224 | 2.501213 | -3.629227 | 2.990134 | 1.341003 | 3.887708 | 0.730 | 2.489651 |
| **HFrTMS-M1 vs Placebo** | -6.677552 | 1.954036 | -9.206408 | 4.430586 | 2.528856 | 4.837064 | 0.601 | 2.638095 |
| **LFrTMS-M1 vs Placebo** | -4.484512 | 1.455331 | -1.266976 | 5.51313 | -3.217536 | 5.683758 | 0.571 | 2.654191 |
| **iTBS-Cerebellum vs Placebo** | -3.0159 | 1.572474 | -14.05724 | 122.6366 | 11.04134 | 122.6478 | 0.928 | 2.315981 |
| **iTBS-M1 vs Placebo** | -.6 | 2.591734 | -14.0306 | 164.5257 | 13.4306 | 164.5455 | 0.935 | 2.315834 |

HFrTMS, high-frequency repetitive transcranial magnetic stimulation; LFrTMS, low-frequency repetitive transcranial magnetic stimulation; iTBS, intermittent theta-burst stimulation; M1, primary motor cortex.

**Supplementary Table 6.** Local consistency test for MEP amplitude.

| Side | Direct |  | Indirect |  | Difference |  |  | tau |
| --- | --- | --- | --- | --- | --- | --- | --- | --- |
|  | Coef. | Std. Err. | Coef. | Std. Err. | Coef. | Std. Err. | P>z |  |
| **HFrTMS-M1 vs LFrTMS-M1** | -.6623775 | .6134918 | -.6874427 | .8177719 | .0250652 | 1.022759 | 0.980 | .7667389 |
| **HFrTMS-M1 vs Placebo** | -.8432023 | .504844 | -1.532484 | .9458198 | .6892815 | 1.07127 | 0.520 | .7351407 |
| **LFrTMS-M1 vs Placebo** | -.4260125 | .4017283 | .3517278 | 1.059604 | -.7777403 | 1.133259 | 0.493 | .720953 |
| **iTBS-M1 vs Placebo** | .0982741 | .7713126 | -1.982479 | 63.26319 | 2.080753 | 63.26789 | 0.974 | .6792633 |

HFrTMS, high-frequency repetitive transcranial magnetic stimulation; LFrTMS, low-frequency repetitive transcranial magnetic stimulation; iTBS, intermittent theta-burst stimulation; M1, primary motor cortex.

**Supplementary Table 7.** Summary of adverse events.

| Study | Type of intervention | Adverse events |
| --- | --- | --- |
| Liu et al 2019 | E1: HFrTMS-M1 | 2 cases of dizziness, facial flushing, and numbness of the scalp |
|  | E2: LFrTMS-M1 | No adverse events |
|  | C: Placebo | No adverse events |
| Du et al 2019 | E1: HFrTMS-M1 | 2 cases of transient headache |
|  | E2: LFrTMS-M1 | No adverse events |
|  | C: Placebo | No adverse events |
| Sharma et al 2020 | E: LFrTMS-M1 | 1 case of epilepsy |
|  | C: Placebo | No adverse events |
| Yan et al 2023 | E: LFrTMS-M1 | 2 cases of local pricking pain, 2 cases of nausea and vomiting, 2 cases of local itching, and 1 case of joint pain |
|  | C: Placebo | 3 cases of local pricking pain, 2 cases of nausea and vomiting, 3 cases of local itching, and 2 cases of joint pain |
| Lin et al 2015 | E: LFrTMS-M1 | 1 case of dizziness |
|  | C: Placebo | 1 case of tingling scalp pain |
| Liao et al 2021 | E: iTBS-Cerebellum | 1 case of mild headache |
|  | C: Placebo | No adverse events |
| Chieffo et al 2021 | E: dTMS-M1 | 3 cases of transitory dizziness and 1 case of muscle twitches on shoulders |
|  | C: Placebo | No adverse events |

E, experimental group; C, control group; HFrTMS, high-frequency repetitive transcranial magnetic stimulation; LFrTMS, low-frequency repetitive transcranial magnetic stimulation; iTBS, intermittent theta-burst stimulation; dTMS, deep transcranial magnetic stimulation; M1, primary motor cortex.
